# *Lactobacillus plantarum* HFY05 Attenuates Carrageenan-Induced Thrombosis in Mice by Regulating NF-κB Pathway-Associated Inflammatory Responses

**DOI:** 10.3389/fnut.2022.813899

**Published:** 2022-03-04

**Authors:** Shi Zeng, Ruokun Yi, Fang Tan, Peng Sun, Qiang Cheng, Xin Zhao

**Affiliations:** ^1^Department of Neurosurgery, People's Hospital of Chongqing Banan District, Chongqing, China; ^2^Chongqing Collaborative Innovation Center for Functional Food, Chongqing Engineering Research Center of Functional Food, Chongqing Engineering Laboratory for Research and Development of Functional Food, Chongqing University of Education, Chongqing, China; ^3^Department of Public Health, Our Lady of Fatima University, Valenzuela, Philippines

**Keywords:** lactic acid bacteria, thrombosis, carrageenan, anti-inflammatory, gut flora

## Abstract

In this study, a carrageenan-induced thrombus model was established in mice to observe the ability of *Lactobacillus plantarum* KFY05 (LP-KFY05) to inhibit thrombosis through an NF-κB-associated pathway. Biochemical analysis, microscopical observations, quantitative polymerase chain reactions (qPCR) and western blot analysis were used to examine relevant serum and tissue indexes, and the composition of intestinal microorganisms was determined by examining the abundance of microorganisms in feces. The results showed that LP-KFY05 could markedly reduce the degree of black tail in thrombotic mice; increase the activated partial thromboplastin time (APTT); and decrease the thrombin time (TT), fibrinogen (FIB) level, and prothrombin time (PT). LP-KFY05 could also reduce tumor necrosis factor alpha (TNF-α), interleukin-6 (IL-6), and interleukin-1 beta (IL-1β) levels in sera and renal tissues of thrombotic mice. Hematoxylin and eosin staining showed that LP-KFY05 could alleviate renal tissue lesions and tail vein thrombosis. qPCR results showed that LP-KFY05 could down-regulate nuclear factor kappa-B (NF-κB) p65, IL-6, TNF-α, and interferon γ (IFN-γ) mRNA expression in renal tissues, as well as NF-κB p65, intercellular cell adhesion molecule-1 (ICAM-1), vascular cell adhesion molecule-1 (VCAM-1), and E-selectin mRNA expression in tail vein vascular tissues of thrombotic mice. Western blot analysis showed that LP-KFY05 also down-regulated NF-κB protein expression in renal and tail vein vascular tissues of thrombotic mice. Lastly, LP-KFY05 increased the abundances of *Bacteroidetes, Lactobacillus*, and *Bifidobacterium*, as well as decreased the abundance of *Firmicutes*. These results show that LP-KFY05 can reduce inflammation and inhibit thrombosis in thrombotic mice, and the effects of high concentrations of LP-KFY05 were most pronounced, which were similar to the effects of dipyridamole.

## Introduction

With the improvement of human living standards, great changes have taken place in diets, which have brought about many adverse effects on human health. Cardiovascular and cerebrovascular diseases continue to threaten the lives of humans, especially those of middle-aged adults and the elderly (1). Most cardiovascular and cerebrovascular diseases show no obvious symptoms before onset, and their onset is usually sudden and serious. If they are not treated on time, then they can result in death, with cerebrovascular diseases associating with a very high mortality rate (2). Thrombosis is the main cause of death due to cardiovascular diseases, which includes chronic thrombosis and results in cerebral ischemia, hypoxia, tissue softening, and necrosis (3). Carrageenan can induce an acute inflammatory reaction in the body. After inflammatory factors are released into the blood, they can damage vascular endothelial cells and cause thrombosis. Because the tail in mice contains a single femoral blood vessel, it is very difficult to establish collateral circulation upon embolization, and gradual ischemia and necrosis of the tail tissue usually ensue (4). In this study, we report that carrageenan caused systemic inflammation in mice, resulting in tail vein thrombosis in mice. We also established an animal thrombus model.

Inflammation is a complex defense response to endogenous or exogenous injurious factors. Recent studies have shown that inflammation can induce thrombosis, and thrombosis can promote the development of inflammation. Furthermore, the degree of organ injury after thrombosis depends not only on the initial injury, but also on the degree of the subsequent vascular thrombosis and inflammation (5, 6). In severe cases, thrombosis-induced inflammation can spread throughout the body and damage distal organs, especially the lungs and kidneys, thereby leading to multiple organ dysfunction and death (7). Under the action of inflammatory factors, blood is in a hypercoagulable state, and thus, capable of inducing thrombosis. At the same time, a variety of thrombotic factors can further aggravate the occurrence and the development of inflammation. Monocytes, platelets, macrophages, and endothelial cells are involved in this process, forming a complex network and causing organ damage, especially inflammation, which is also an important influencing factor of cerebral thrombosis (8, 9).

Natural fermented yak yogurt, which is consumed by inhabitants of Qinghai Tibet Plateau in China, is a traditional fermented dairy product with characteristics of local Tibetian foods, and it is superior to general yogurt in terms of nutrition and health benefits (10). It has been reported that the health benefits of yak yogurt are related to the rich lactic acid bacteria contained therein, and the types of lactic acid bacteria in yak yogurt are mainly influenced by the living habits of herdsmen in various regions, milking and fermentation equipment, fermentation temperature, time, and other factors. Therefore, the lactic acid bacteria isolated from yak yogurt in pastoral areas are very different from the commonly-used commercial lactic acid bacteria isolated from general yogurt (11, 12). Studies have demonstrated that lactic acid bacteria isolated from yak yogurt show better colonization in the intestine, as well as good preventive and interventive effects on colitis and gastritis (13, 14) and good regulatory effects on hyperlipidemia (15). As such, these lactic acid bacteria have good biological activity in the body. In this study, a type of lactic acid bacteria (*Lactobacillus plantarum* HFY05, LP-HFY05) isolated from naturally-fermented yak yogurt by our team was studied. Using a mouse tail vein thrombosis model, we observed the interventive effects of LP-HFY05 on thrombosis by examining its ability to regulate inflammation in the body, so as to identify a new way to prevent thrombosis and to assist in the elimination of thrombosis, as well as to reduce the risk of diseases such as cerebral thrombosis. Dipyridamole is an antithrombotic drug, and in this study, it was used as a positive control and compared with LP-HFY05.

## Materials and Methods

### Experimental Strain

LP-HFY05 was isolated from natural fermented yak yogurt in Hongyuan County, Sichuan Province, Qinghai Tibet Plateau, China. The strain was identified and stored at the China General Microbiological Culture Collection Center (Beijing, China). The deposit number was CGMCC 16635. During the experiment, LP-HFY05 was resuscitated for use.

### *In vivo* Experiment

Fifty male six-week-old specific pathogen free (SPF) Institute of Cancer Research (ICR) mice weighing 23 ± 2 g were purchased from the Experimental Animal Center of Chongqing Medical University (<city>Chongqing </city>, China). All experiments complied with the proper treatment of experimental animals, and other experimental procedures complied with the ethical requirements of experimental animals. The housing conditions were as follows: a temperature of 20 ± 1°C, a relative humidity of 30–40%, free access to food and water, 12 h:12 h cycles of light:dark, and an adaptive feeding cycle of 7 days. Fifty ICR mice were randomly divided into five groups with ten mice in each group as follows: normal group, model group, dipyridamole (positive control) group, LP-HFY05 low-concentration (LP-HFY05-L) group, and LP-HFY05 high-concentration (LP-HFY05-H) group. The normal-group mice were intraperitoneally injected with 0.01 mL/g normal saline solution every day, and the other groups of mice were intraperitoneally injected with 0.01 mL/g carrageenan solution (Shanghai Sunbio Co., Ltd.) at a final concentration of 0.2% for 10 days (16, 17). A previous study has demonstrated that to prevent diseases, the human body should be supplemented with at least 6 × 10^10^ probiotics per day, and according to this measurement, this was converted to an experimental measurement of 10^9^ CFU/kg per day. At the same time, this study showed that two concentrations (10^8^ and 10^9^ CFU/kg) of lactic acid bacteria could induce an upward trend of positive effects, and these two concentrations were also used in this study (18). Mice in the dipyridamole group were given dipyridamole at a final concentration of 20 mg/kg per day, whereas mice in LP-HFY05-L and LP-HFY05-H groups were given LP-HFY05 at a final concentration of 10^8^ CFU/kg and 10^9^ CFU/kg per day, respectively, and the intragastric administration of dipyridamole and LP-HFY05 spanned 10 days. After 10 days, the tail thrombus length of mice in each group was recorded.

### Four-Item Hemagglutination Test

The blood collected before the mice were killed was placed in a centrifuge tube containing sodium citrate, centrifuged (1,500 rpm, 4°C, 10 min) to collect the plasma, and the four-item hemagglutination test was performed to measure the activated partial thromboplastin time (APTT), thrombin time (TT), fibrinogen level (FIB), and prothrombin time (PT) with a semi-automatic hemagglutination instrument (PUN-2048A, Beijing Pulang New Technology Co., Ltd., Beijing, China).

### Determination of the Inflammatory Factor Index

The whole blood samples were centrifuged (4,000 rpm, 4°C, 10 min) to collect the serum. At the same time, 0.1 g of renal tissue was accurately weighed, and then 0.9 mL of normal saline was added to the renal tissue for homogenization. The homogenized tissue was centrifuged (4,000 rpm, 4°C, 10 min), and the supernatant was collected. The inflammatory indexes of TNF-α, IL-6, and IL-1β in sera and renal tissues were measured with detection kits (Shanghai Enzyme Link Biotechnology Co., Ltd., Shanghai, China) as previously described (19).

### Histopathological Observation

The mice were euthanized by cervical dislocation and dissected. After dissection, renal and tail tissues of mice were collected and fixed in 10% formalin. After 48 h of dehydration, the tissue samples were embedded in paraffin, sectioned, and stained with hematoxylin eosin (H&E). The pathological changes were observed with a light microscope (BX43, Olympus, Tokyo, Japan) as previously described (20).

### Determination of MRNA Expression in Mouse Tail Vein and Renal Tissues

In brief, 0.2 g of tail vein and renal tissues were accurately weighed, 9 mL of normal saline was added to the samples for homogenization, and then 0.5 mL of TRIzol reagent (Invitrogen, Carlsbad, CA, USA) was added to extract RNA from the samples. The RNA absorbance values were measured at 260 nm and 280 nm by super differential spectrophotometry using the Nano-300 instrument (Hangzhou Allsheng Instruments Co., Ltd., Zhejiang, China). The RNA purity and concentration were calculated, and the RNA concentration was adjusted to 1 μg/μL. Thereafter, cDNA was prepared by reverse transcription using 1 μL of cDNA, 10 μL of SYBR Green PCR Master Mix, 7 μL of sterile distilled water (Thermo Fisher Scientific, Waltham, MA, USA), and 1 μL each of forward and reverse primers (Table 1). The reaction was carried out in a quantitative PCR instrument using conditions as follows: 95°C for 60 s and 95°C for 15 s, followed by 40 cycles of 55°C for 30 s; 72°C for 35 s; 95°C for 30 s; and 55°C for 35 s. GAPDH was used as the internal control, and the 2^−ΔΔCt^ method was used to analyze the target genes as previously described (21).

**Table 1:** Primer sequences in this experiment.

**Gene**	**Forward sequence**	**Reverse sequence**
*NF-κB p65*	5′-GAGGCACGAGGCTCCTTTTCT-3′	5′-GTAGCTGCATGGAGACTCGAACA-3′
*ICAM-1*	5′-TCCGCTACCATCACCGTGTAT-3′	5′-TAGCCAGCACCGTGAATGTG-3′
*VCAM-1*	5′-TTGGGAGCCTCAACGGTACT-3′	5′-GCAATCGTTTTGTATTCAGGGGA-3′
*E-selectin*	5′-ATAACGAGACGCCATCATGC-3′	5′-TGTCCACTGCCCTTGTGC-3′
IL-6	5′-ATGAAGTTCCTCTCTGCAA-3′	5′-AGTGGTATCCTCTGTGAAG-3′
TNF-α	5′-ATGGGGGGCTTCCAGAA-3	5′-CCTTTGGGGACCGATCA-3′
IFN-γ	5′-GCTTTGCAGCTCTTCCTCAT-3′	5′-GTCACCATCCTTTTGCCAGT-3′
*Total bacteria*	5'-ACTCCTACGGGAGGCAGCAGT-3'	5'-ATTACCGCGGCTGCTGGC-3'
*Firmicutes*	5'-GCGTGAGTGAAGAAGT-3'	5'-CTACGCTCCCTTTACAC-3'
*Bacteroidetes*	5'-ACGCTAGCTACAGGCTTAACA-3'	5'-ACGCTACTTGGCTGGTTCA-3'
textitLactobacillus	5'-CACCGCTACACATGGAG-3'	5'-AGCAGTAGGGAATCTTCCA-3'
*Bifidobacteria*	5'-TCGCGTCYGGTGTGAAAG-3'	5'-CCACATCCAGCRTCCAC-3'
*GAPDH*	5′-TGACCTCAACTACATGGTCTACA-3′	5′-CTTCCCATTCTCGGCCTTG-3′

### Determination of Microbial MRNA Expression in Mouse Feces

In brief, 1 g of mouse feces was accurately weighed, and 9 mL of normal saline was added to the samples for homogenization. The mRNA expression of microorganisms in mouse feces was determined according to the aforementioned tissue mRNA determination method, so as to detect the composition of microorganisms in feces.

### Western Blot Analysis

In brief, 100 mg of tail vein and renal tissues were obtained and washed three times with pre-cooled PBS. Thereafter, 1 mL of pre-cooled protein lysis buffer was added, and the tissues were homogenized and centrifuged (10,000 rpm, 4°C, 15 min). After centrifugation, the intermediate layer was the total protein fraction. The bicinchonic acid detection kit (Nanjing Jiancheng Bioengineering Institute, Nanjing, China) was used to determine the protein concentration. Sodium dodecyl sulfate-polyacrylamide gel electrophoresis was employed to separate the heat-denatured proteins, and the proteins were transferred to a polyvinylidene fluoride membrane. The membrane was blocked with 5% skim milk for 1 h, incubated with an NF-κB primary antibody (Thermo Fisher Scientific) overnight at 4°C, and washed with 1 × TBST on the following day. The membrane was incubated with a secondary antibody (Cell Signaling Technology Inc., Danvers, MA, USA) for 1 h at 25°C. Finally, the iBright Western Blot imaging system (Thermo Fisher Scientific) was used to detect the immunoreactive proteins, and β-actin was used as an internal control as previously described (21).

### Statistical Analysis

Three parallel determinations were carried out for all experiments. The results were expressed as the average value, and the standard deviation was calculated. The experimental results were expressed as the average value ± standard deviation. Student–Newman–Keuls (SNK) multiple-range test was used to determine whether there were significant differences between the groups (*P* < 0.05). SAS 9.1 software (SAS Institute, Inc., Cary, NC, USA) was utilized for statistical analyses.

## Results

### Length of Tail Vein Thrombosis in Mice

**Figure 1:**
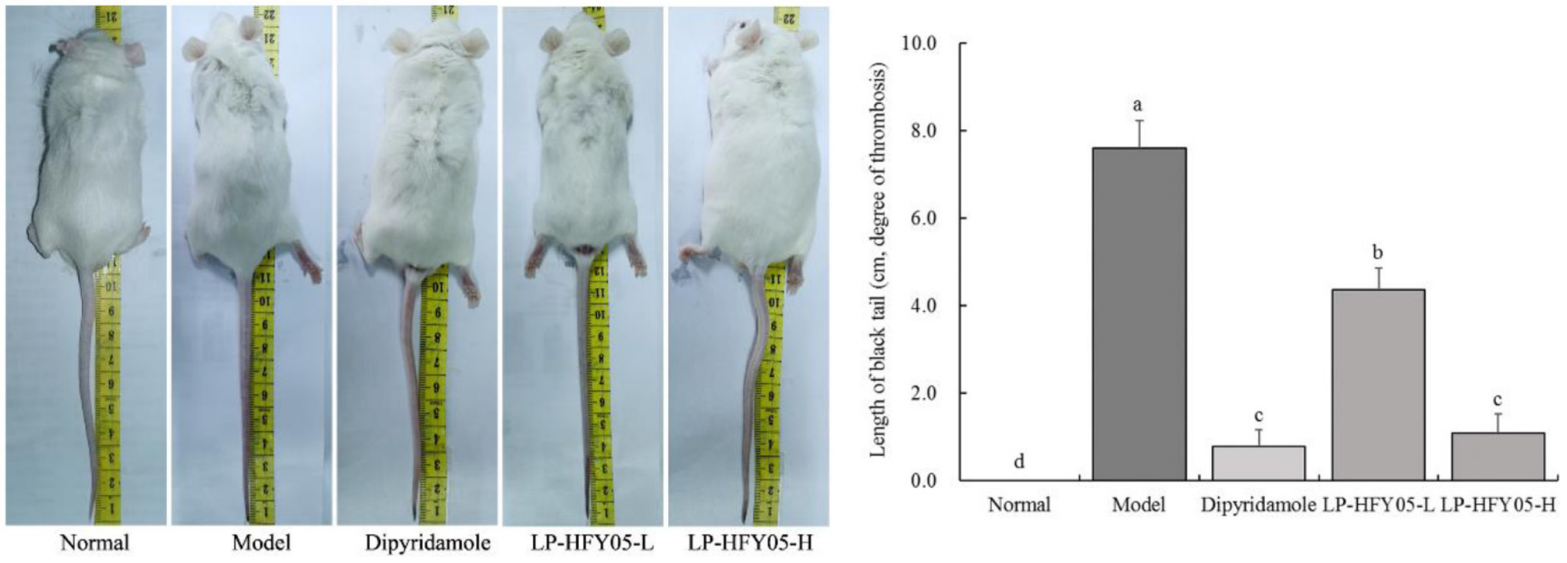
Tail thrombus of mice in each group. ^a−*d*^ Mean values with different letters in the different bars were significantly different (*P* < 0.05) according to the SNK multiple-range test. ^a−*d*^ Mean values with different letters in the different bars were significantly different (*P* < 0.05) according to the SNK multiple-range test; b, c, and d indicate significance vs the model group; the same letter of c indicates that there was no significant difference (*P* > 0.05) between the two groups.

Figure 1 shows that after the intraperitoneal injection of carrageenan, a black tail appeared at the tail tip of mice in each group, that is, tail vein thrombosis was observed. The length of the black tail in the model group was the longest at 7.6 ± 0.6 cm, which was significantly longer than that in the other groups (*P* < 0.05). The length of the black tail in the LP-HFY05-H group was the shortest at 1.1 ± 0.4 cm, similar to the dipyridamole group (0.8 ± 0.4 cm), and significantly shorter than that of the LP-HFY05-L group (4.4 ± 0.5 cm, *P* < 0.05).

### APTT, TT, FIB, and PT of Mice

As shown in Table 2, the APTT of normal mice was significantly higher (*P* < 0.05) than that of the other groups, whereas the TT, FIB, and PT were significantly lower (*P* < 0.05) than those of the other groups. The model group showed the opposite findings; the APTT was significantly lower (*P* < 0.05) than that of the other groups, whereas the TT, FIB, and PT were significantly higher (*P* < 0.05) than those of the other groups. The APTT of the LP-HFY05-H group was significantly higher (*P* < 0.05) than that of the LP-HFY05-L group, whereas the TT, FIB, and PT were significantly lower (*P* < 0.05) than those of the LP-HFY05-L group. The APTT, TT, FIB, and PT of the LP-HFY05-H group were similar to those of the dipyridamole group, and there was no significant difference between the two groups (*P* > 0.05).

**Table 2:** Activated partial thromboplastin time (APTT), thrombin time (TT), fibrinogen (FIB), and prothrombin time (PT) of mice.

**Group**	**APTT (s)**	**TT (s)**	**FIB (g/L)**	**PT (s)**
Normal	162.2 ± 4.3^d^	50.7 ± 5.0^d^	75.2 ± 4.1^d^	7.6 ± 1.1^d^
Model	89.1 ± 4.9^a^	82.1 ± 3.8^a^	114.8 ± 6.4^a^	20.2 ± 1.8^a^
Dipyridamole	129.3 ± 4.2^c^	61.1 ± 5.2^c^	84.5 ± 3.5^c^	10.3 ± 1.2^c^
LP-HFY05-L	110.0 ± 43.9^b^	74.1 ± 6.4^b^	93.2 ± 4.8^b^	14.8 ± 1.7^b^
LP-HFY05-H	126.3 ± 5.1^c^	64.6 ± 3.6^c^	86.2 ± 4.4^bc^	11.4 ± 1.3^c^

a−d *Mean values with different letters in the different bars are significantly different (P <0.05) according to the SNK multiple-range test; b, c, and d indicate significance vs the model group; the same letter of c indicates that there was no significant difference (P > 0.05) between the two groups*.

### TNF-α, IL-6, and IL-1β Levels in Mice

**Figure 2:**
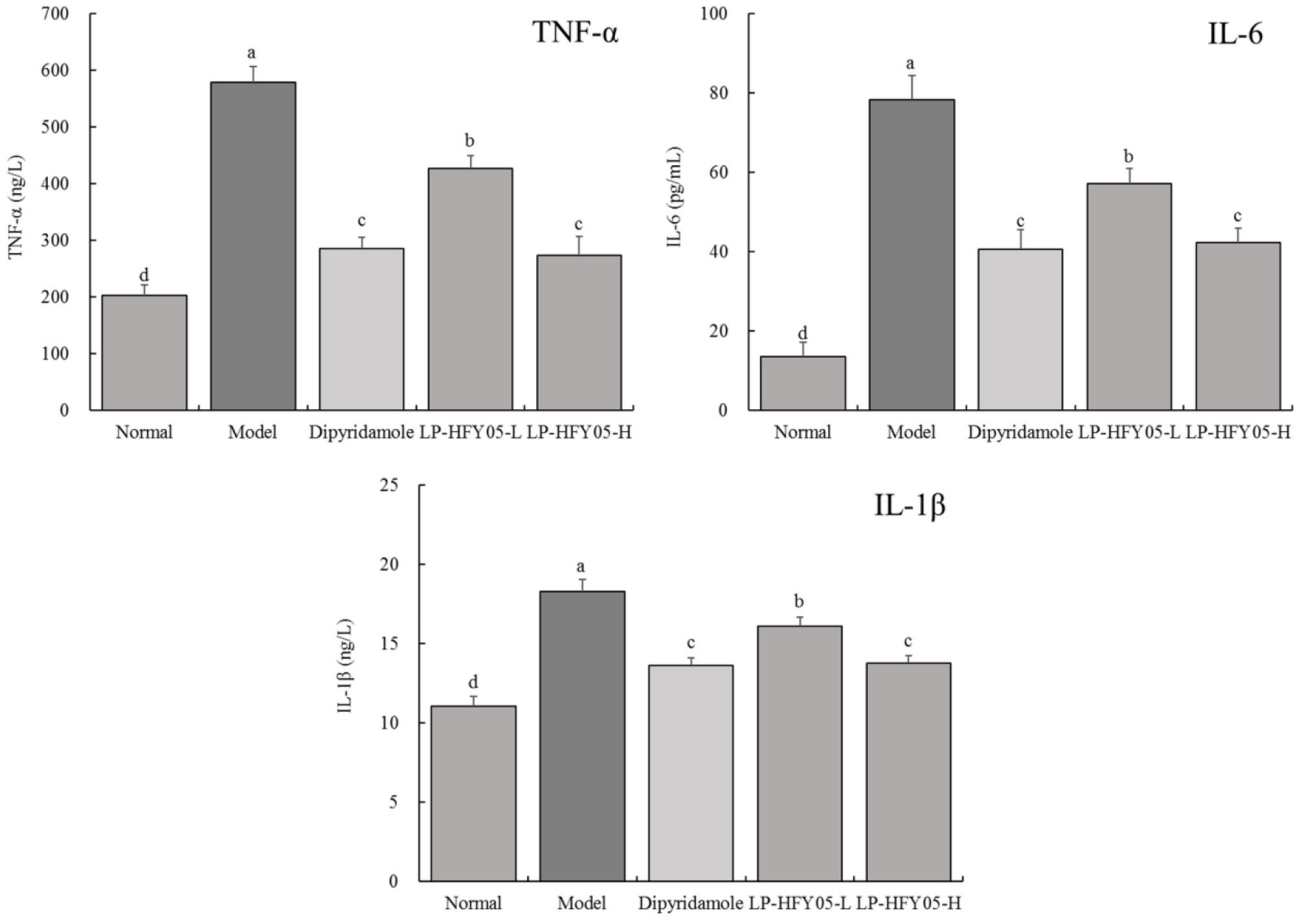
TNF-α, IL-6, and IL-1β levels in serum of mice with thrombosis. ^a−*d*^ Mean values with different letters in the different bars were significantly different (*P* < 0.05) according to the SNK multiple-range test; b, c, and d indicate significance vs the model group; the same letter of c indicates that there was no significant difference (*P* > 0.05) between the two groups.

**Figure 3:**
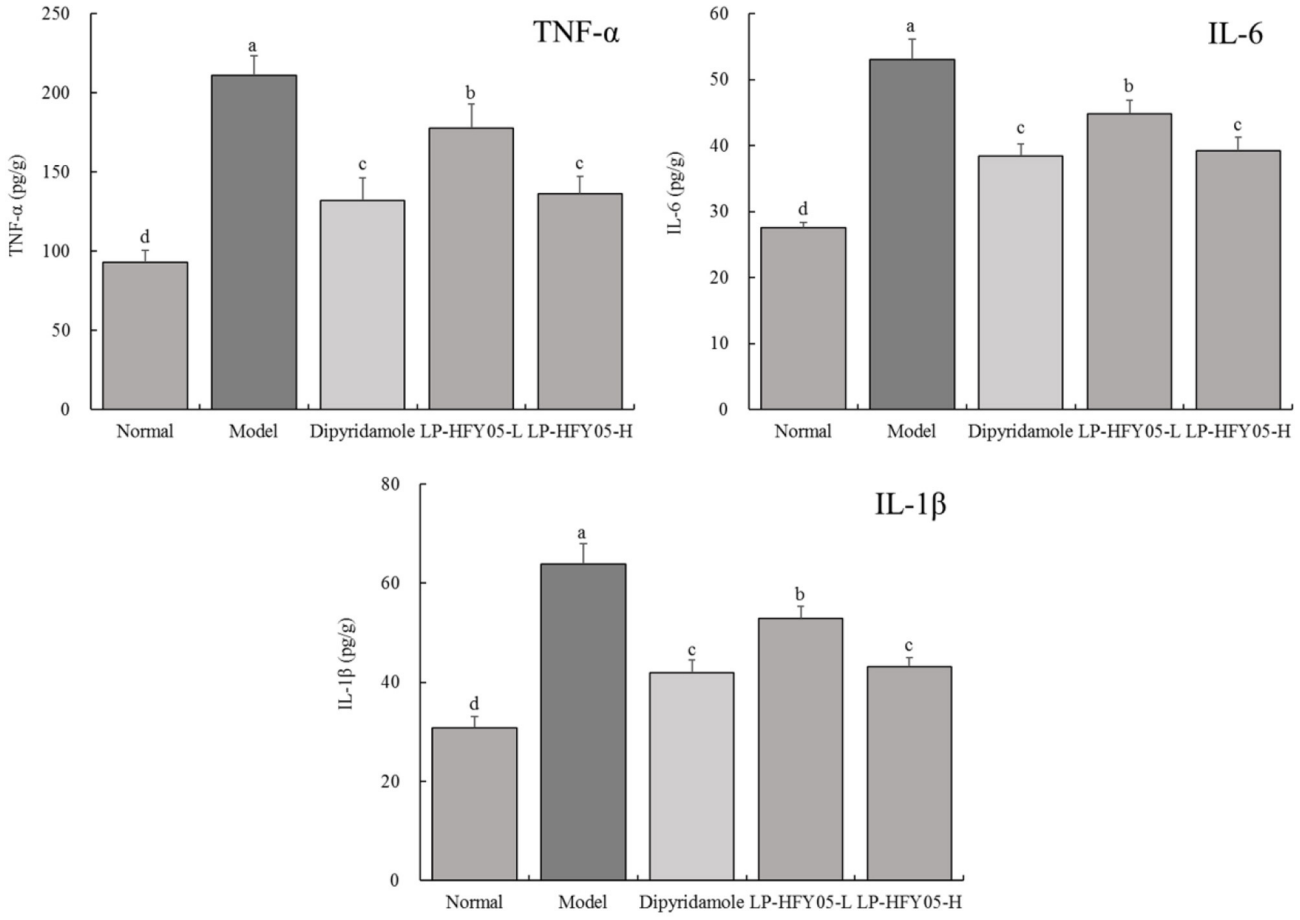
TNF-α, IL-6, and IL-1β levels in renal tissues of mice with thrombosis. ^a−*d*^ Mean values with different letters in the different bars were significantly different (*P* < 0.05) according to the SNK multiple-range test; b, c, and d indicate significance vs the model group; the same letter of c indicates that there was no significant difference (*P* > 0.05) between the two groups.

As shown in Figures 2, 3, TNF-α, IL-6, and IL-1β levels in serum and renal tissues of normal, dipyridamole, LP-HFY05-H, LP-HFY05-L, and model groups showed a trend from low to high. TNF-α, IL-6, and IL-1β levels in dipyridamole and LP-HFY05-H groups were significantly lower than those in the LP-HFY05-L group (*P* < 0.05), but there was no significant difference between the dipyridamole group and LP-HFY05-H group (*P* > 0.05).

### Pathological Observations in Mice

**Figure 4:**
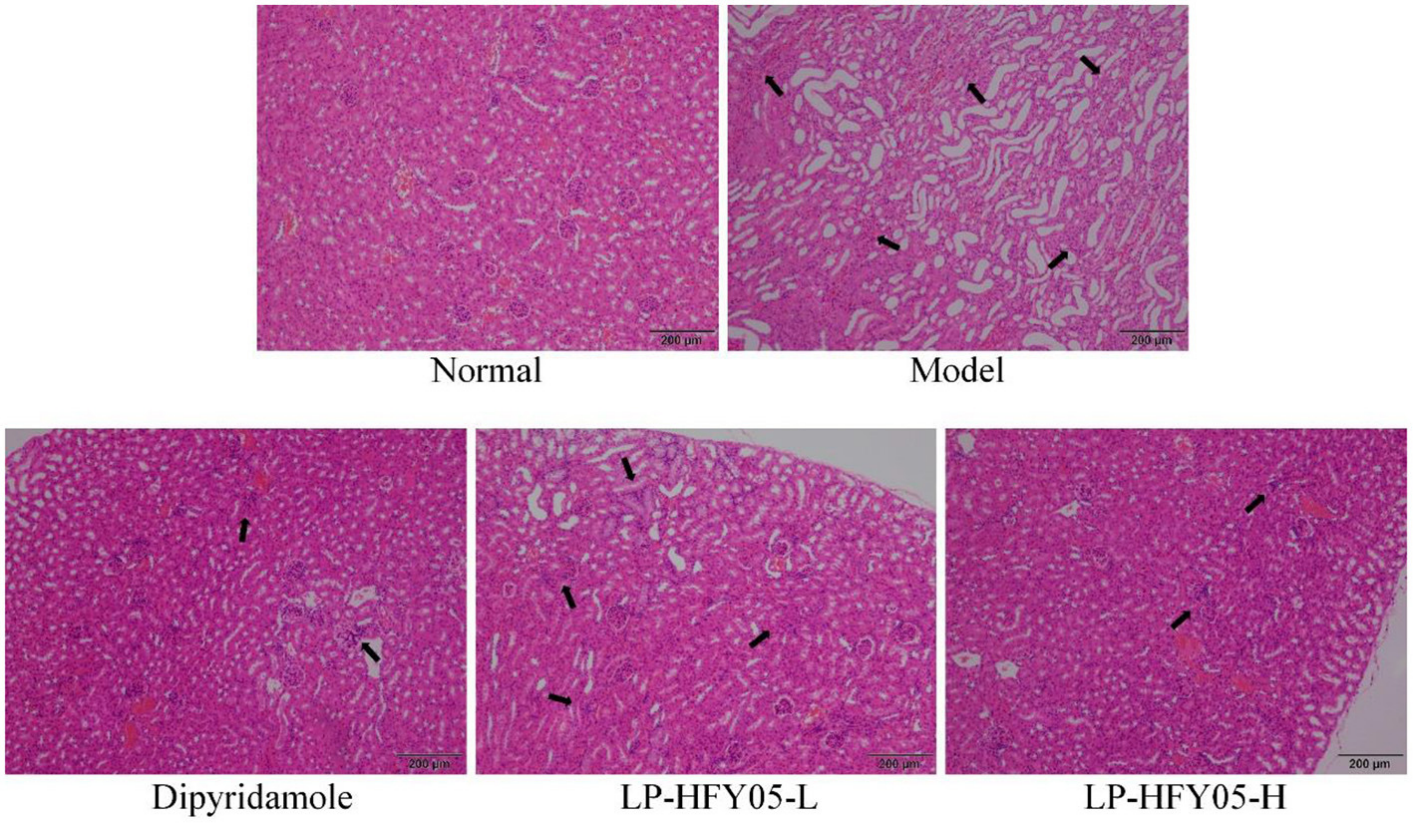
Image of hematoxylin and eosin-stained tail vein vessels in mice. The arrow points to damaged cells.

As shown in Figure 4, the tail vein vessels in the normal group were round and clean, and the vessel wall was smooth. In the model group, there was leukocyte infiltration, inflammatory exudation, bloody lesions, platelet aggregation, and thrombosis associating with the vessel wall of the tail vein. Both LP-HFY05 and dipyridamole could reduce the extent of the lesions in the tail vein vessels in mice, and the effects of LP-HFY05-H and dipyridamole were better than those of LP-HFY05-L, and the effects of LP-HFY05-H and dipyridamole were similar.

**Figure 5:**
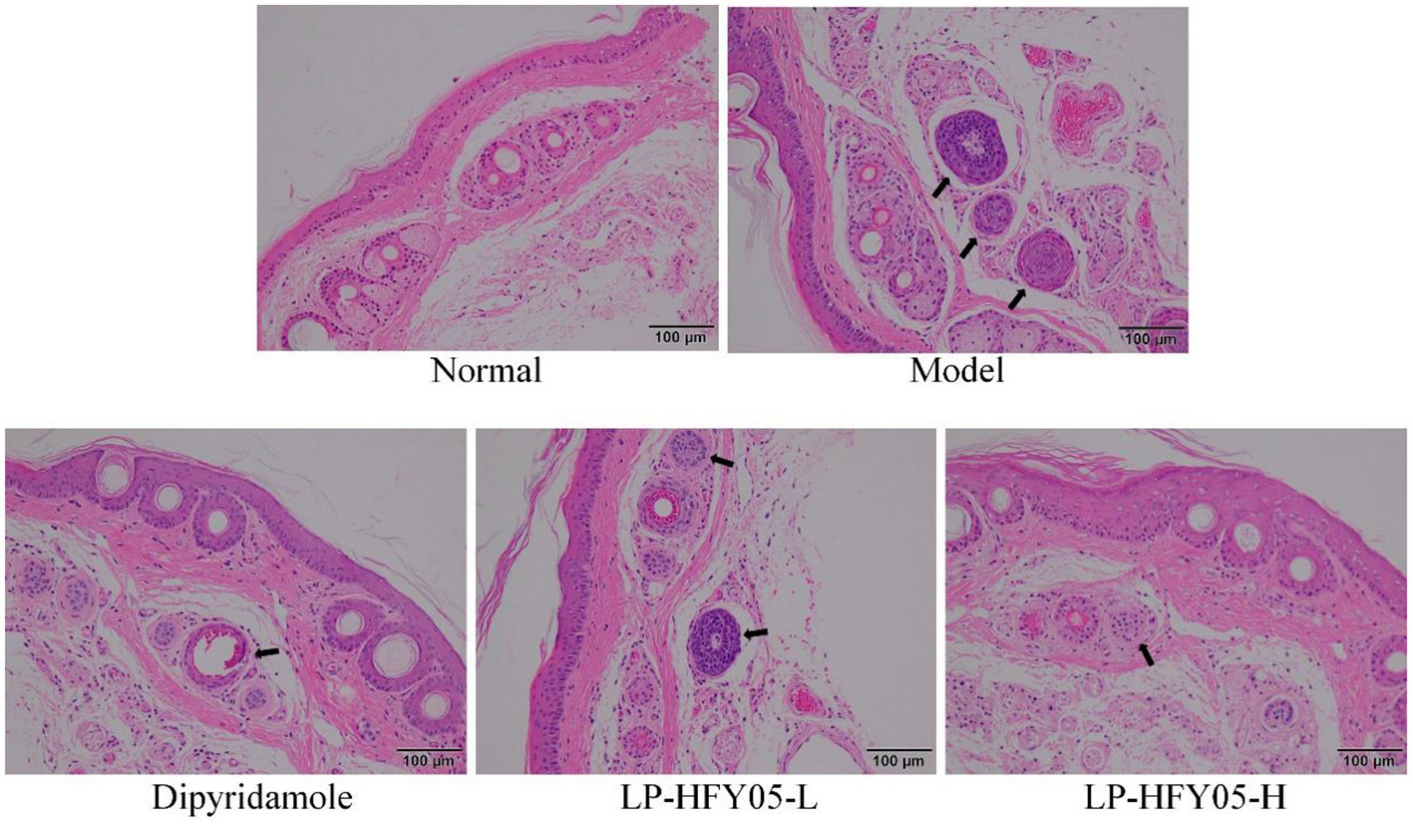
Image of hematoxylin and eosin-stained renal tissue in mice. The arrow points to the thrombotic site.

The hematoxylin and eosin staining results are shown in Figure 5. The renal tissues of mice in the model group showed significantly enlarged and diseased glomeruli, interstitial edema, inflammatory cell infiltration, tubular epithelial cell proliferation, degenerating glomerular epithelial cell swelling, edema-like degeneration, and luminal narrowing. There were no changes in the renal tissues of mice in the normal group, and LP-HFY05-H and dipyridamole could alleviate the inflammatory lesions in the renal tissues caused by superior thrombus and reverse the renal tissue morphology so that it was similar to the normal group. LP-HFY05-L could also reduce the extent of the lesions in the renal tissues of mice with thrombosis mice to some extent.

### mRNA Expression of NF-κB p65, IL-6, TNF-α, and IFN-Γ in Mouse Renal Tissues

**Figure 6:**
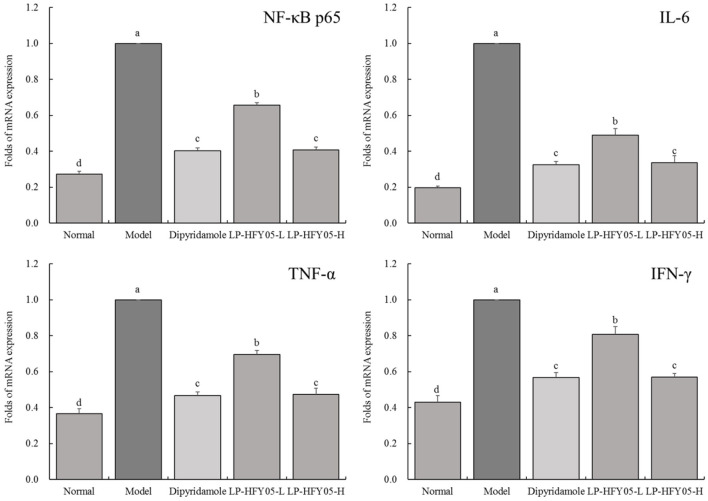
mRNA expression of NF-κB p65, IL-6, TNF-α, and IFN-γ in renal tissues of mice with thrombosis. ^a−*d*^ Mean values with different letters in the different bars were significantly different (*P* < 0.05) according to the SNK multiple-range test; b, c, and d indicate significance vs the model group; the same letter of c indicates that there was no significant difference (*P* > 0.05) between the two groups.

As shown in Figure 6, the mRNA levels of NF-κB p65, IL-6, TNF-α, and IFN-γ in the renal tissues of mice in the normal group were significantly lower (*P* < 0.05) than those in the other groups, and the mRNA levels of NF-κB p65, IL-6, TNF-α, and IFN-γ in the model group were significantly higher (*P* < 0.05) than those in the other groups. LP-HFY05 and dipyridamole could down-regulate NF-κB p65, IL-6, TNF-α, and IFN-γ expression in renal tissues of mice with thrombosis, and the effects of LP-HFY05-H and dipyridamole were similar, and the effects of both were significantly better (*P* < 0.05) than those of LP-HFY05-L.

### mRNA Expression of NF-κB p65, ICAM-1, VCAM-1, and E-Selectin in Mouse Tail Vein

**Figure 7:**
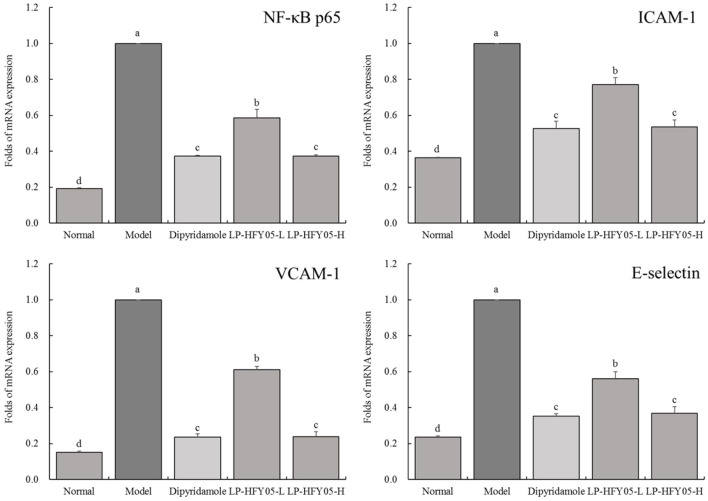
mRNA expression of NF-κB p65, ICAM-1, VCAM-1, and E-selectin in tail vein tissues of mice with thrombosis. ^a−*d*^ Mean values with different letters in the different bars were significantly different (*P* < 0.05) according to the SNK multiple-range test; b, c, and d indicate significance vs the model group; the same letter of c indicates that there was no significant difference (*P* > 0.05) between the two groups.

As shown in Figure 7, the mRNA levels of NF-κB p65, ICAM-1, VCAM-1, and E-selectin in mice tail vein vessels in the model group were the highest. LP-HFY05-L, LP-HFY05-H, and dipyridamole significantly (*P* < 0.05) down-regulated NF-κB p65, ICAM-1, VCAM-1, and E-selectin expression in the tail vein of thrombosed mice. However, these expression patterns were not significantly different (*P* < 0.05) between LP-HFY05-H and dipyridamole groups, and mRNA levels in LP-HFY05-H and dipyridamole groups were higher than those in the normal group.

### Protein Expression of NF-κB in Mouse Tail Vein and Renal Tissues

**Figure 8:**
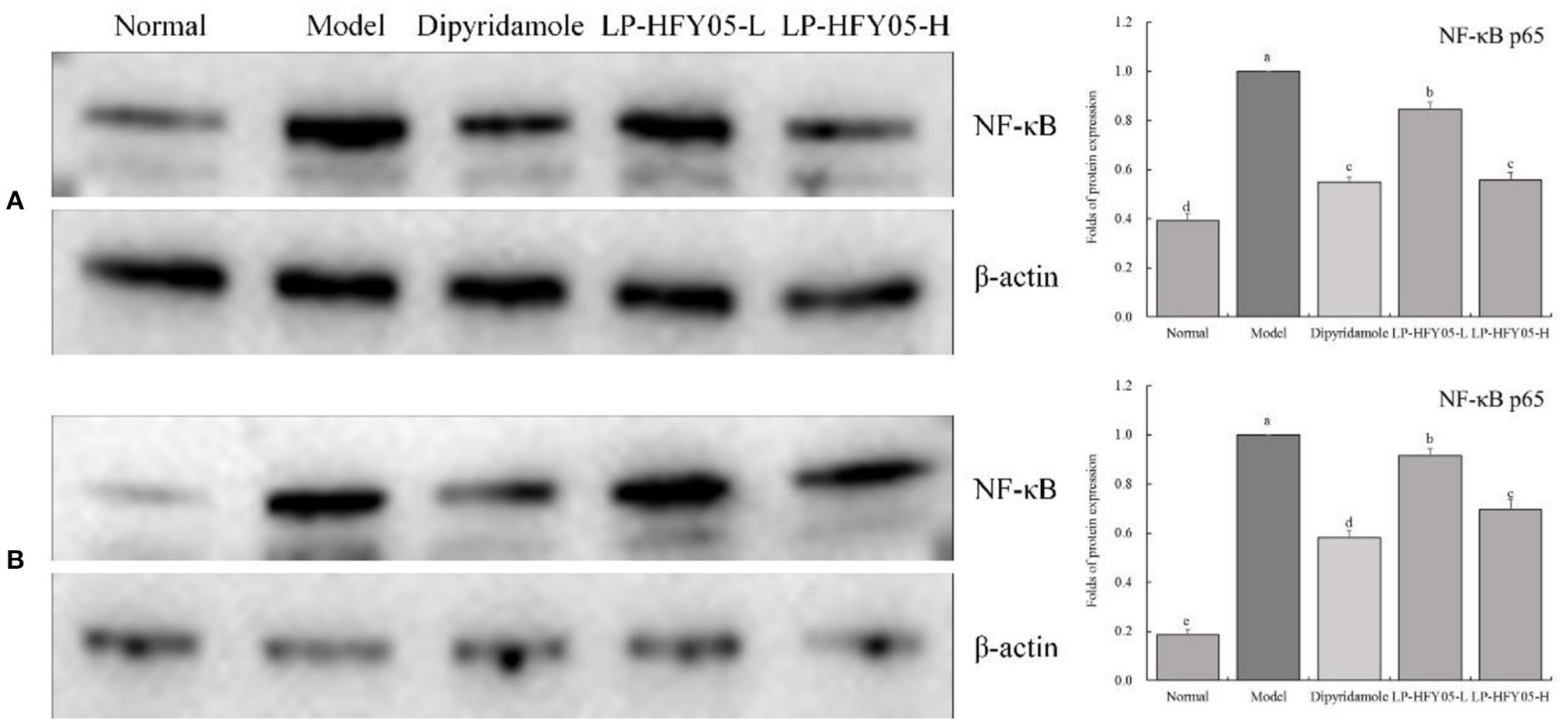
Protein expression of NF-κB in renal **(A)** and tail vein **(B)** tissues of mice with thrombosis. ^a−*d*^ Mean values with different letters in the different bars were significantly different (*P* < 0.05) according to the SNK multiple-range test; **(A)**: b, c, and d indicate significance vs the model group; the same letter of c indicates that there was no significant difference (*P* > 0.05) between the two groups; **(B)**: b, c, d, and e indicate significance vs the model group.

As shown in Figure 8, NF-κB protein expression in tail vein and renal tissues of mice in the model group was the highest. On the contrary, NF-κB protein expression was the lowest in the normal group. Compared with the model group, LP-HFY05-H and dipyridamole could down-regulate NF-κB protein expression more than LP-HFY05-L.

### mRNA Expression of Firmicutes, Bacteroidetes, Lactobacillus, and Bifidobacteria in Mouse Feces

**Figure 9:**
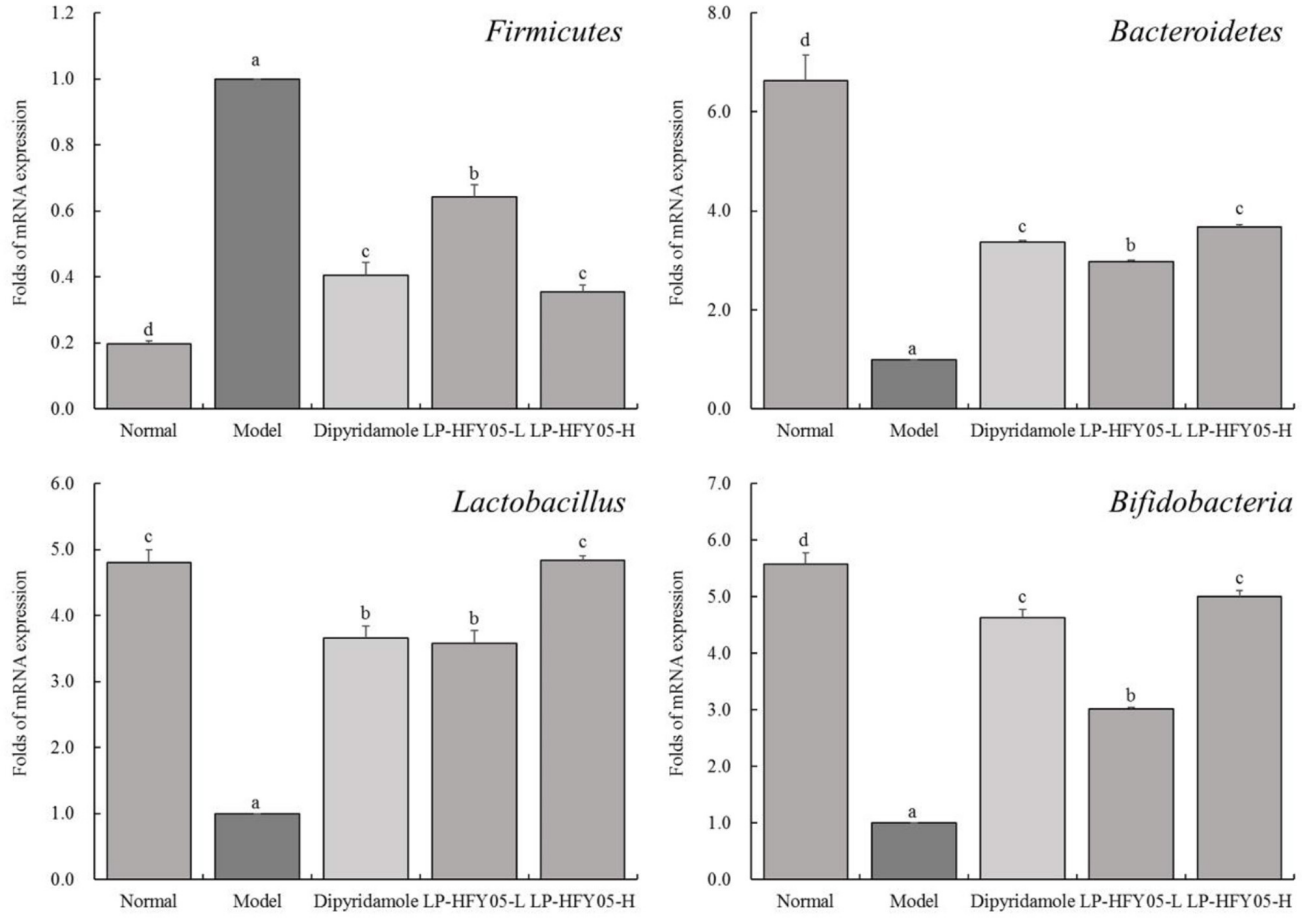
The mRNA expression of *Firmicutes, Bacteroidetes, Lactobacillus*, and *Bifidobacteria* in mouse feces. ^a−*d*^ Mean values with different letters in the different bars are significantly different (*P* < 0.05) according to SNK multiple-range test; *Firmicutes, Bacteroidetes* and *Bifidobacteria*: b, c and d are for significance vs model group; the same letter of c indicates that there is no significant difference (*P* > 0.05) between the two groups; *Lactobacillus*: b and c are for significance vs model group; the same letter of b or c indicates that there is no significant difference (*P* > 0.05) between the two groups.

As shown in Figure 9, the mRNA expression of *Firmicutes* in feces of normal mice was significantly lower (*P* < 0.05) than that of the other groups, whereas the mRNA expression of *Bacteroidetes* and *Bifidobacterium* was significantly higher (*P* < 0.05) than that of the other groups. After the induction of thrombosis, the mRNA expression of *Firmicutes* in feces of mice in the model group was the highest, whereas the expression of *Bacteroidetes, Lactobacillus*, and *Bifidobacteria* was the lowest. LP-HFY05 and dipyridamole reduced *Firmicutes* expression and enhanced *Bacteroidetes* and *Bifidobacteria* expression in the feces of mice with thrombosis mice. In addition, *Lactobacillus* expression in normal and LP-HFY05-H groups was not significantly different (*P* > 0.05) but significantly higher than that in the other groups (*P* < 0.05).

## Discussion

Carrageenan can induce thrombosis-related intravascular inflammation in the tail of mice, resulting in mixed thrombi filling in the venules, arterioles, and capillaries and causing ischemic necrosis of the tail tissue. The black tails of mice were observed intuitively (15). Therefore, the extent of the black tail in mice is an important experimental index to intuitively judge the degree of thrombosis. This study also proved that carrageenan could form black tail in mice. Dipyridamole and LP-HFY05 could reduce the black tail caused by thrombosis, and the effects of high-concentrations of LP-HFY05 were better, which were similar to the effects of the common drug dipyridamole.

The detection of four hemagglutination parameters (PT, APTT, TT, and FIB) is of great significance in the diagnosis of blood diseases associating with abnormal coagulation (22). During thrombosis, several coagulation factors are involved, resulting in the prolongation of the PT, whereas the loss of coagulation factors leads to the shortening of the APTT. At the same time, under the action of thrombin, FIB is continuously transformed into fibrin (the main component of the thrombus), and it maintains the body's blood in a hypercoagulable state. When the fibrin content in the blood is too high, fibrinolysis is enhanced and fibrin degradation products are increased, resulting in the prolongation of the TT (23, 24). In this study, dipyridamole and LP-HFY05 could control the four indexes of hemagglutination, indicating that dipyridamole and LP-HFY05 could intervene thrombosis.

Inflammation can induce thrombosis, and thrombosis can further aggravate the development of inflammation. This vicious circle is called the “thrombo-inflammatory response” (25). After inflammation, monocytes, macrophages, lymphocytes, and endothelial cells can be activated by toll like receptor (TLR) to promote the production of TNF-α, IL-6, and other inflammatory mediators. Among them, TNF-α not only plays an important role in the coordination of immune events, but also plays a key role in connecting thrombosis and inflammation (26). Firstly, as the initiating factor of the inflammatory cascade, TNF-α regulates downstream NF-κB and MAPK signaling pathways through its receptor to promote the release of IL-6, IL-8, IL-1, and other inflammatory factors by macrophages, thereby inducing the activation of macrophages, lymphocytes, and other cells, and aggravating vascular endothelial injury and deep venous thrombosis (27). Meanwhile, TNF-α promotes the release of IL-1β from endothelial cells. This leads to vascular endothelial cell injury, platelet adhesion, and thrombosis. TNF-α, IL-1β, and other cytokines not only reduce the transcription and expression of thrombomodulin (TM) in endothelial cells and weaken its anticoagulant activity, but also induce the expression of vasoconstrictors, cause vasoconstriction, and promote thrombosis (28). Therefore, TNF-α, IL-6, and IL-1β control the inflammatory response and avoid thrombosis. At the same time, they also inhibit thrombosis. The inflammatory response is involved in the development of thrombosis. TNF-α and IFN-γ are typical inflammatory factors, and a regulation of their levels is important in the regulation of thrombosis (29). Monocytes and T cells have roles in the maintenance of immunity; in addition, they develop into tissue macrophages and dendritic cells and participate in the pathophysiological process of procoagulation (anticoagulation) (30). The activation of TNF-α and IFN-γ signaling pathways can regulate monocytes and T cells (31). Therefore, LP-HFY05 plays a role in regulating monocytes and T cells by controlling TNF-α expression, thereby regulating thrombosis.

The kidney is an important organ for excreting human metabolites and water. If there is a problem with the kidneys, then toxins in the human body will not be excreted (32). The involvement of the kidneys in thrombosis has a serious impact on metabolism and detoxification of the body. The kidneys experience an inflammatory reaction that can result in renal failure, which is life-threatening. Therefore, thrombosis in other organs of the body will be reflected by the physiological changes in the kidneys (33). Through pathological observations, this study found that inflammatory lesions appeared in renal tissues after tail thrombosis in mice, which confirms that tail thrombosis in mice can involve renal lesions. Dipyridamole and LP-HFY05 could inhibit these lesions, and LP-HFY05 with probiotic potential showed effects similar to those of dipyridamole. At the same time, the detection of inflammatory cytokines and mRNA expression also confirmed that thrombosis could affect the inflammatory response in the kidneys, and LP-HFY05 could regulate inflammation in this organ.

NF-κB is a key mediator of the inflammatory response in deep venous thrombosis, and NF-κB destroys the balance between coagulation and fusion by mediating the interactions between endothelial cells and platelets, as well as the inflammatory reaction, and induces thrombosis (34). ICAM-1 mediates adhesion between cells and between cells and the extracellular matrix, where it plays an important role in the development of inflammation (35). VCAM-1 can negatively regulate platelet adhesion and aggregation and induce the inflammatory response at the thrombus site (36). E-selectin can mediate local adhesion between leukocytes and endothelial cells under blood flow, induce inflammatory injury in endothelial cells, increase permeability in endothelial cells, and accelerate leukocyte exudation (37). The NF-κB signaling pathway is the central link in a variety of inflammatory responses. In the activated state, it up-regulates the expression of pro-inflammatory factors, and the inflammatory response can be activated by the NF-κB pathway, which activates endothelial cells and leads to the increased expression of adhesion molecules and cytokines, such as ICAM-1, VCAM-1, and E-selectin, thereby further activating NF-κB. To amplify the inflammatory reaction, platelet aggregation and coagulation must occur to create the hypercoagulable state (38). In this study, thrombosis led to the inflammatory reaction in the mouse tail vein. The expression of ICAM-1, VCAM-1, and E-selectin in the NF-κB signaling pathway was significantly different from that in the normal state. Both dipyridamole and LP-HFY05 could regulate NF-κB p65, ICAM-1, VCAM-1, and E-selectin expression, which prompts us to conclude that they have good thrombotic inhibitory effects.

Thrombosis is a major phenomenon in the pathogenesis of stroke. Thrombosis secondary to plaque rupture on the basis of atherosclerosis is the most common cause of stroke. Under pathological conditions, endotoxemia occurs after the cell wall components of intestinal flora enter the blood circulation, thereby causing systemic chronic inflammation. Presently, it is believed that inflammation plays an important role in thrombosis (39, 40). Inflammation caused by an intestinal flora imbalance is often accompanied by the hypercoagulable state. Inflammation can increase the content of coagulation factors and reduce the activity of plasminogen. The human intestinal microbial community is mainly composed of *Firmicutes* and *Bacteroidetes*, accounting for more than 90% of the intestinal flora. When the abundance of *Firmicutes* is higher than that of *Bacteroides* in the intestine, the blood viscosity increases, the possibility of coagulation increases, and the probability of thrombosis greatly increases (41). *Lactobacillus* and *Bifidobacterium* are probiotics with good activity, which can effectively help the body metabolize and excrete toxic substances, regulate blood circulation, reduce inflammation caused by various factors, and reduce the possibility of thrombosis (42). Healthy intestinal flora can reduce the risk of thrombosis by regulating platelet function and changing coagulation function (43). This study also confirmed that LP-HFY05 could enhance the abundance of *Bacteroidetes* and *Bifidobacteria*, as well as supplement the abundance of *Lactobacillus* in the intestine of mice, and reduce the abundance of *Firmicutes*, so as to inhibit inflammation, promote blood circulation, and inhibit thrombosis. In particular, the side effects of dipyridamole included headache, dizziness, nausea, vomiting, diarrhea, and other adverse reactions. However, LP-HFY05, as a lactic acid bacteria isolated from food, had no side effects. As such, it has better application prospects.

## Conclusion

**Figure 10:**
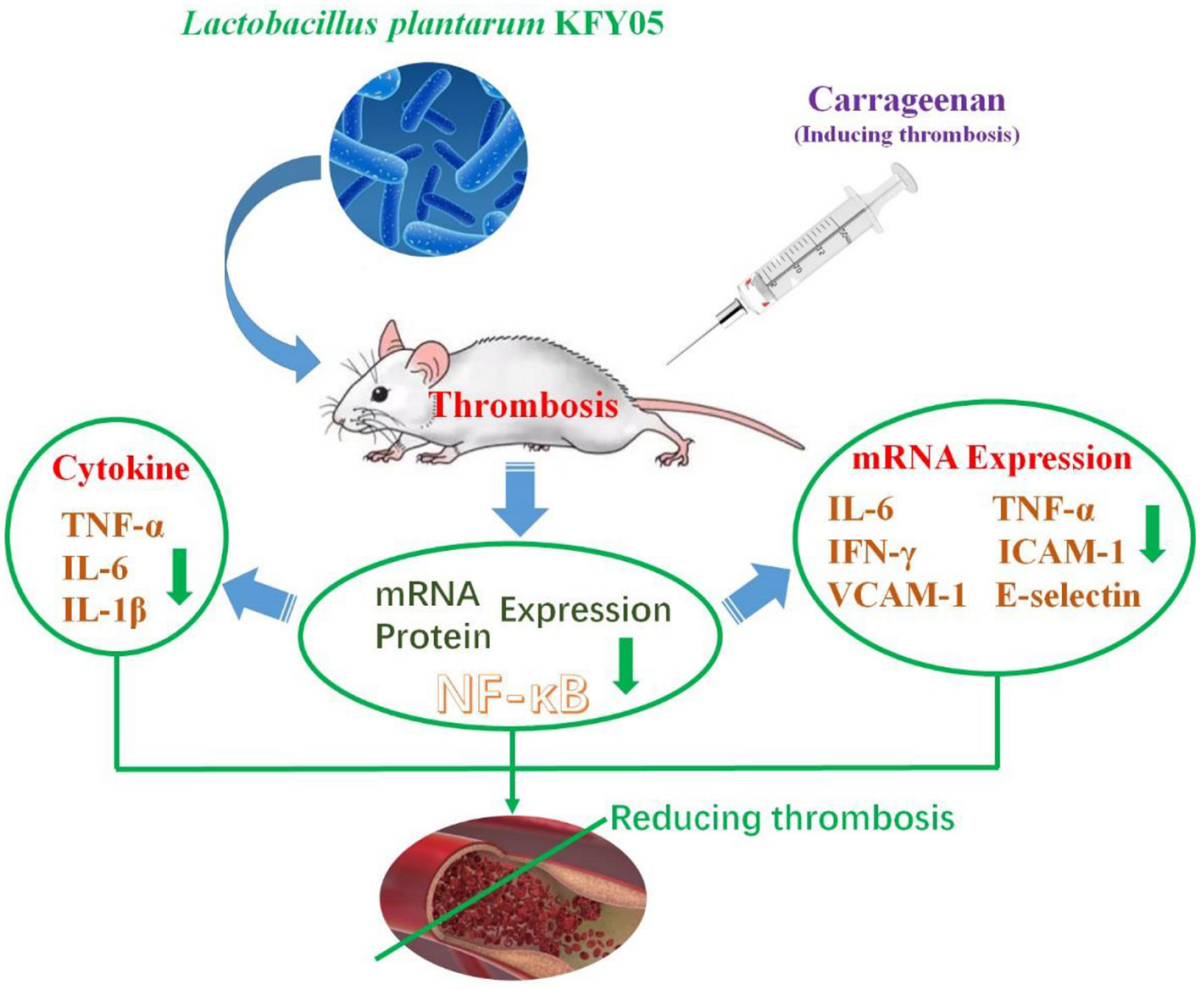
Schematic diagram of the mechanism of *Lactobacillus plantarum* HFY05 on thrombosis by regulating inflammatory response.

In this study, the newly discovered *Lactobacillus plantarum* LP-HFY05 was studied, and its inhibitory effect on thrombosis in mice was investigated through animal experiments. The results showed that LP-HFY05 could effectively regulate the extent of black tail and hemagglutination in thrombotic mice. It could also regulate inflammatory cytokine levels in sera and renal tissues, thereby exerting its inhibitory effects on inflammation (Figure 10). Further results showed that LP-HFY05 could regulate the expression of component proteins in the NF-κB pathway to reduce thrombosis by regulating pro-inflammatory cytokines. At the same time, this study showed that LP-HFY05 regulated the intestine of mice and rendered the intestinal microbial environment healthier, so as to maintain the overall health of the body, reduce the degree of inflammation, and play an inhibitory role in thrombosis. The results show that LP-HFY05 has good inhibitory effects on experimental thrombosis. However, this study still lacks human data, and further clinical studies are needed.

## Data Availability Statement

The raw data supporting the conclusions of this article will be made available by the authors, without undue reservation.

## Ethics Statement

The animal study was reviewed and approved by the protocol for these experiments was approved by the Ethics Committee of Chongqing Collaborative Innovation Center for Functional Food (2021050001B), Chongqing, China. The experimental process was in accordance with 2010/63/EU directive.

## Author Contributions

XZ designed research and supervised the study. SZ and RY wrote the manuscript and interpreted the data. FT and PS analyzed and interpreted the data. All authors agreed to be held accountable for the content herein and approved the final version of the manuscript.

## Funding

This research was funded by the General Program of the Natural Science Foundation of Chongqing (cstc2021jcyjmsxmX0070), China.

## Conflict of Interest

The authors declare that the research was conducted in the absence of any commercial or financial relationships that could be construed as a potential conflict of interest.

## Publisher's Note

All claims expressed in this article are solely those of the authors and do not necessarily represent those of their affiliated organizations, or those of the publisher, the editors and the reviewers. Any product that may be evaluated in this article, or claim that may be made by its manufacturer, is not guaranteed or endorsed by the publisher.
