# Supplementary figures and images for: Lactobacillus plantarum HFY05 Attenuates Carrageenan-Induced Thrombosis in Mice by Regulating NF-κB Pathway-Associated Inflammatory Responses
Source: Front Nutr. 2022 Mar 4;9:813899. doi: 10.3389/fnut.2022.813899 (PMC8931398; doi:10.3389/fnut.2022.813899)

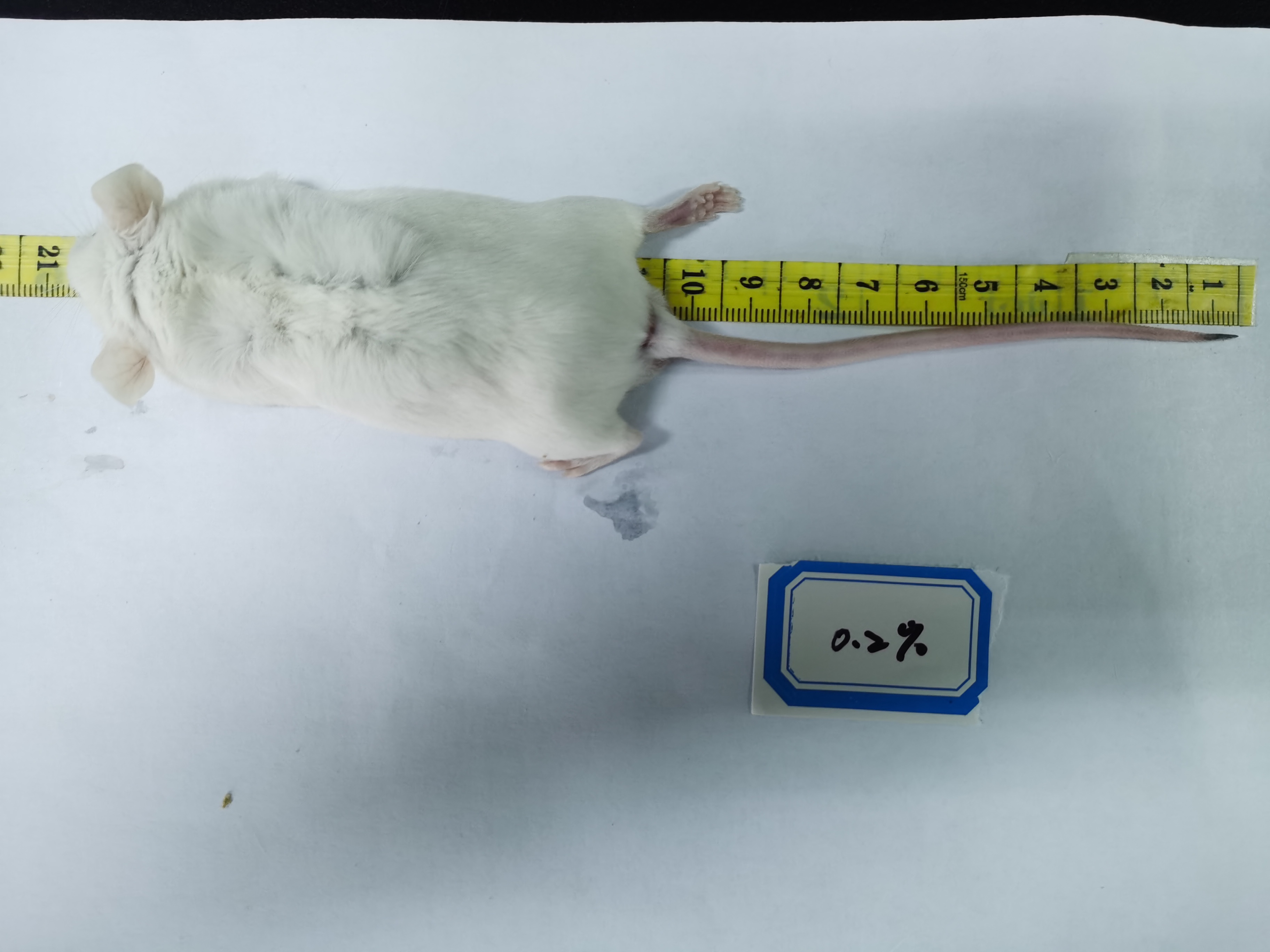

Supplement: Supplementary file 1 [file Data_Sheet_1.ZIP › Figure 1/dipyridamole.jpg]

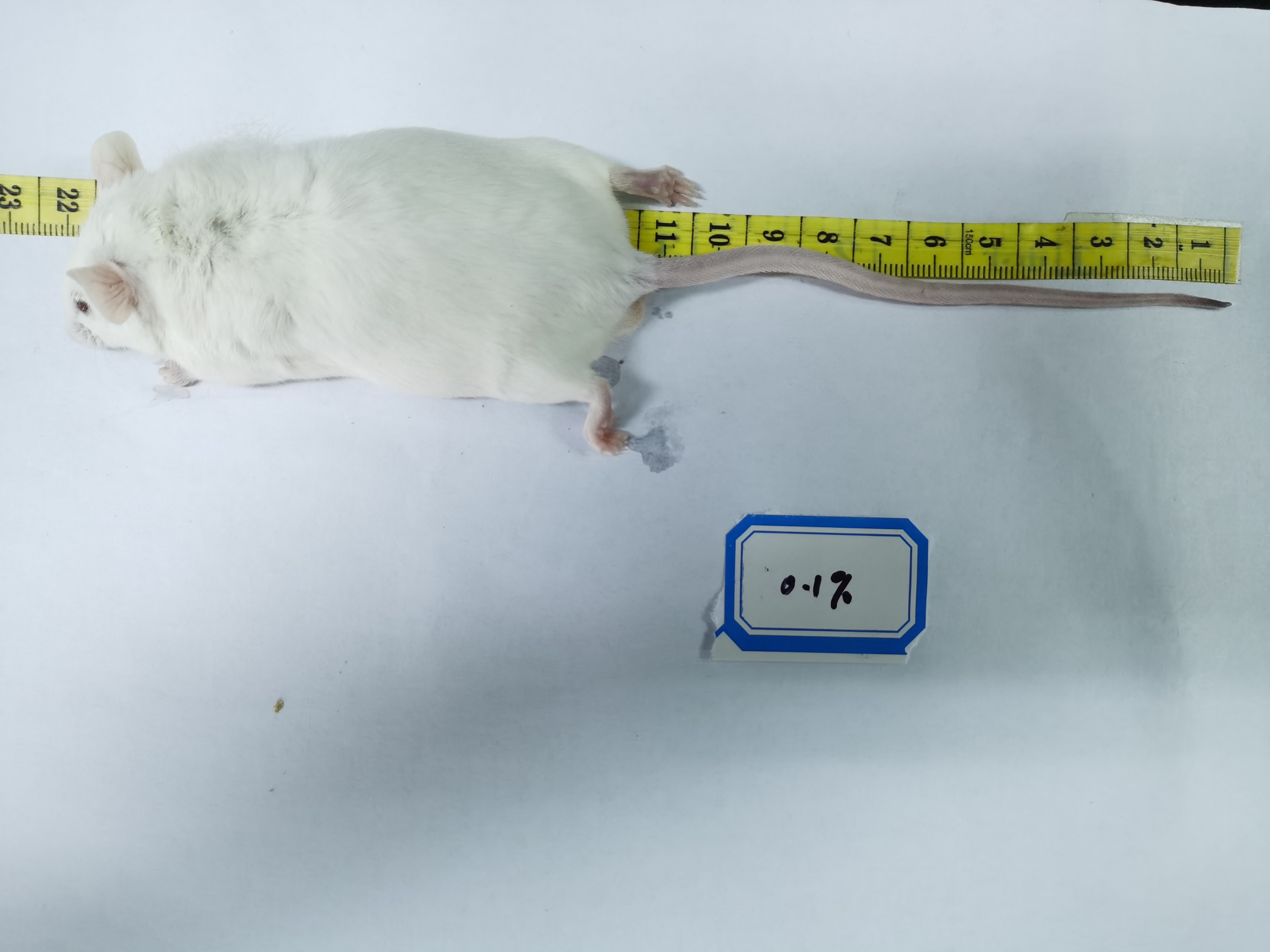

Supplement: Supplementary file 1 [file Data_Sheet_1.ZIP › Figure 1/LP-HFY05-H.jpg]

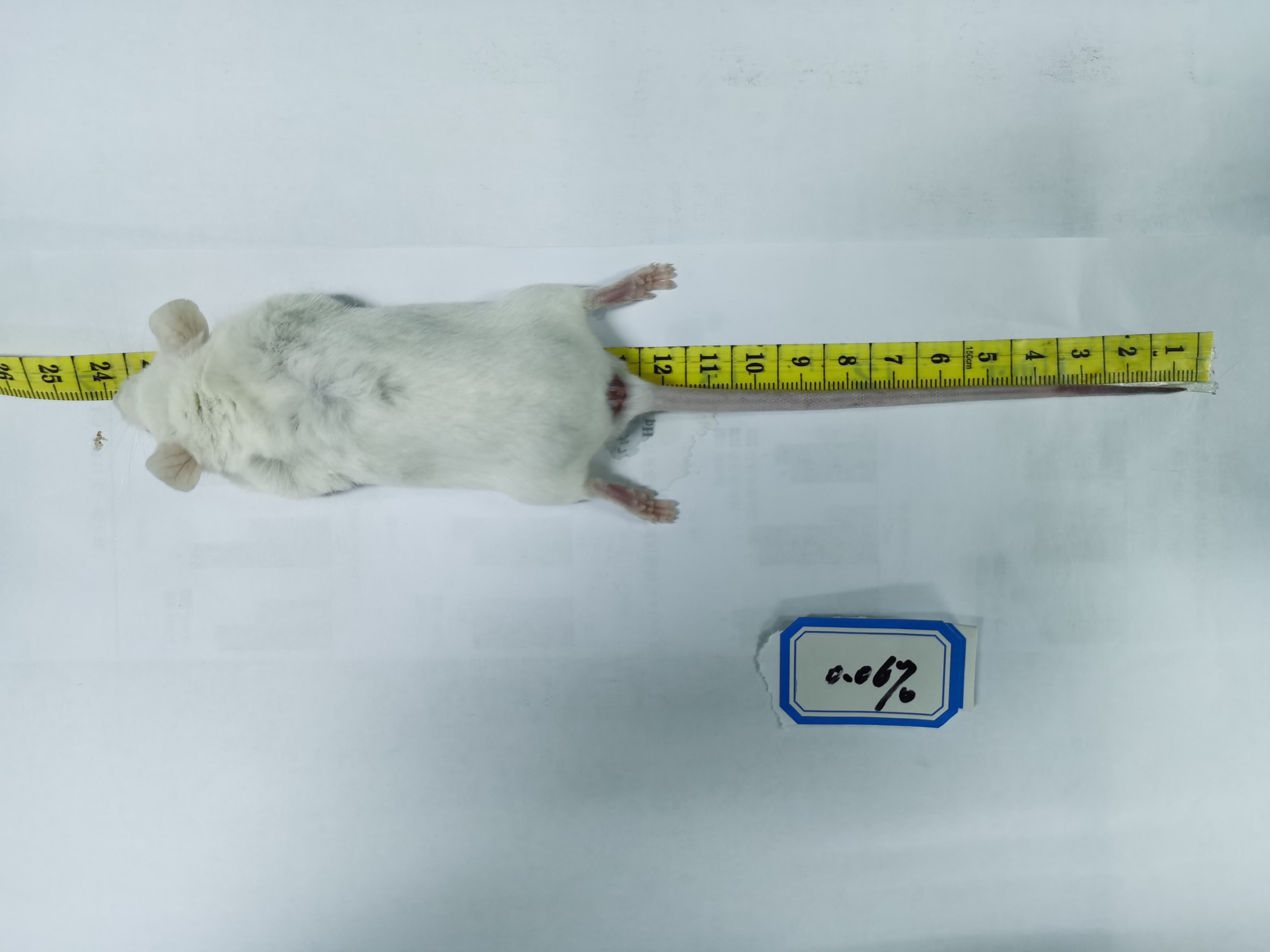

Supplement: Supplementary file 1 [file Data_Sheet_1.ZIP › Figure 1/LP-HFY05-L.jpg]

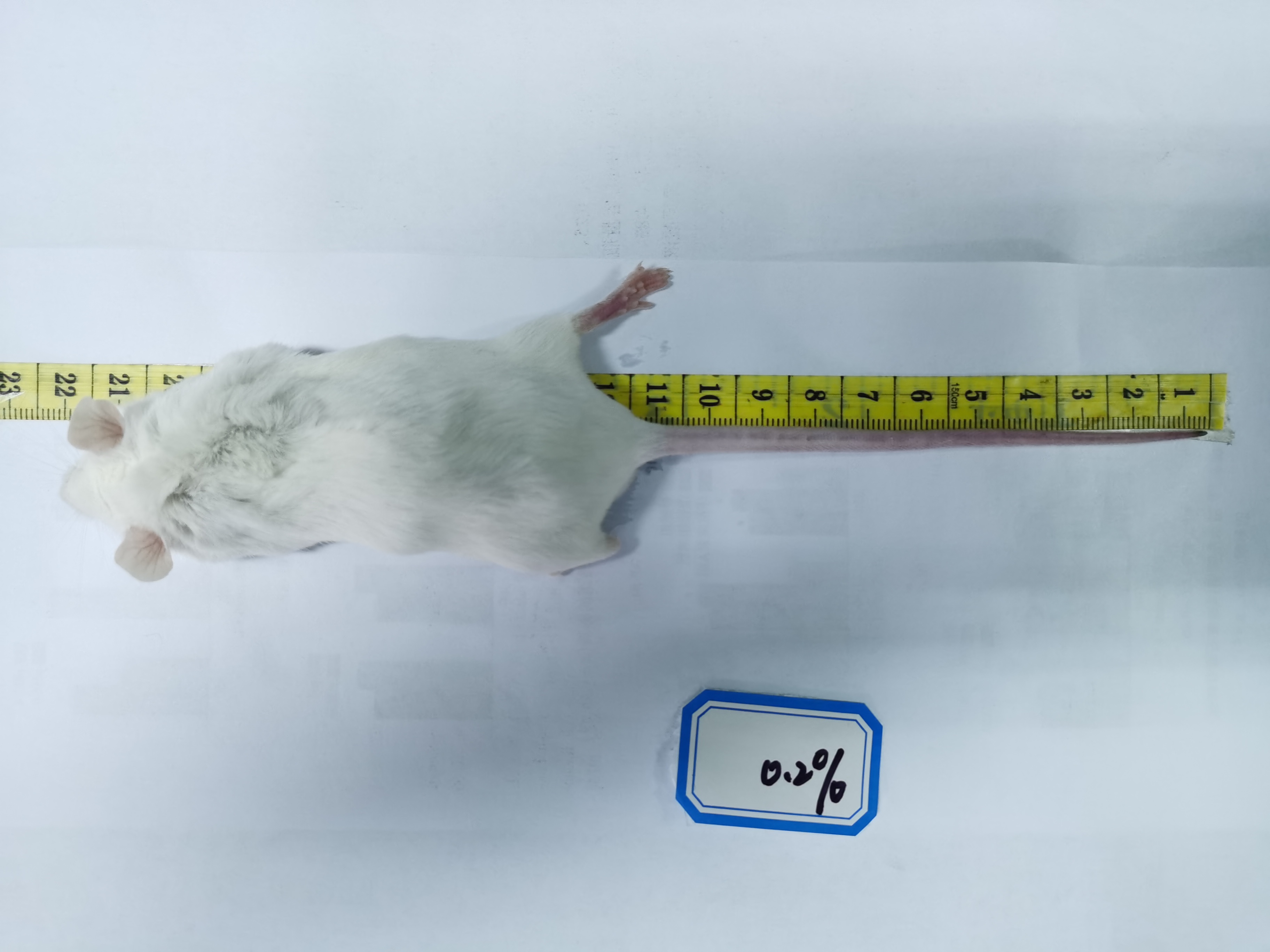

Supplement: Supplementary file 1 [file Data_Sheet_1.ZIP › Figure 1/model.jpg]

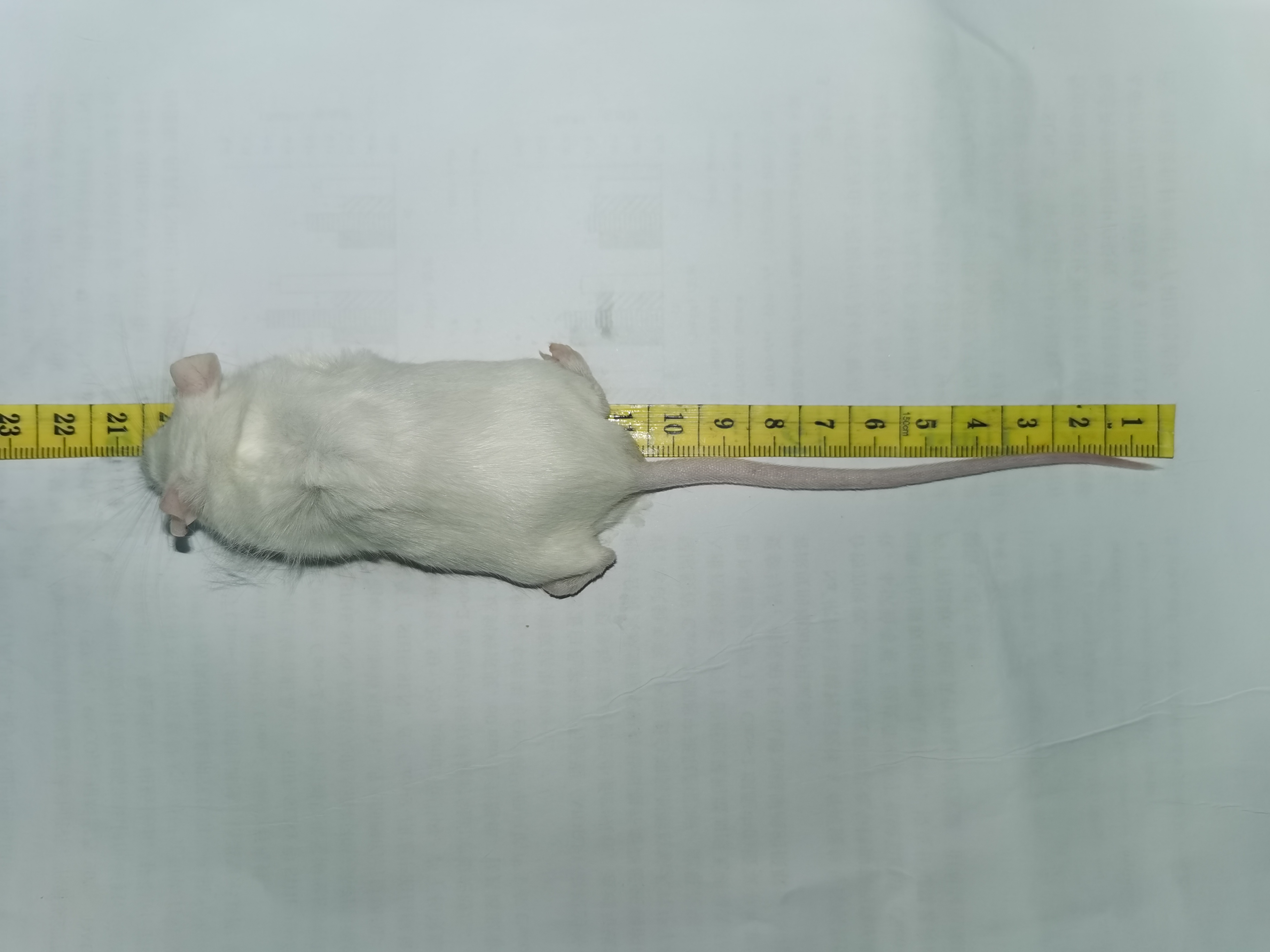

Supplement: Supplementary file 1 [file Data_Sheet_1.ZIP › Figure 1/normal.jpg]

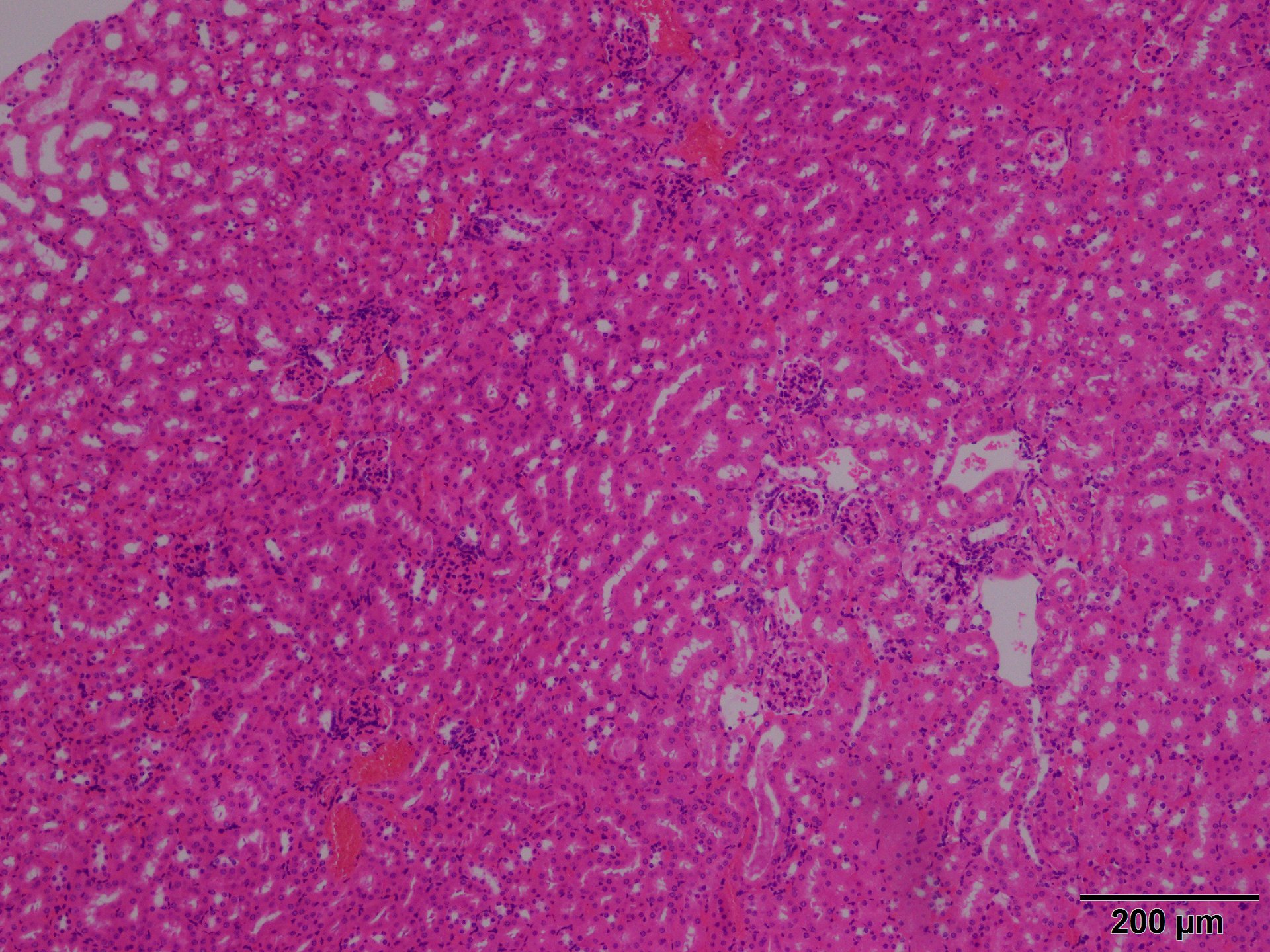

Supplement: Supplementary file 1 [file Data_Sheet_1.ZIP › Figure 4/dipyridamole.jpg]

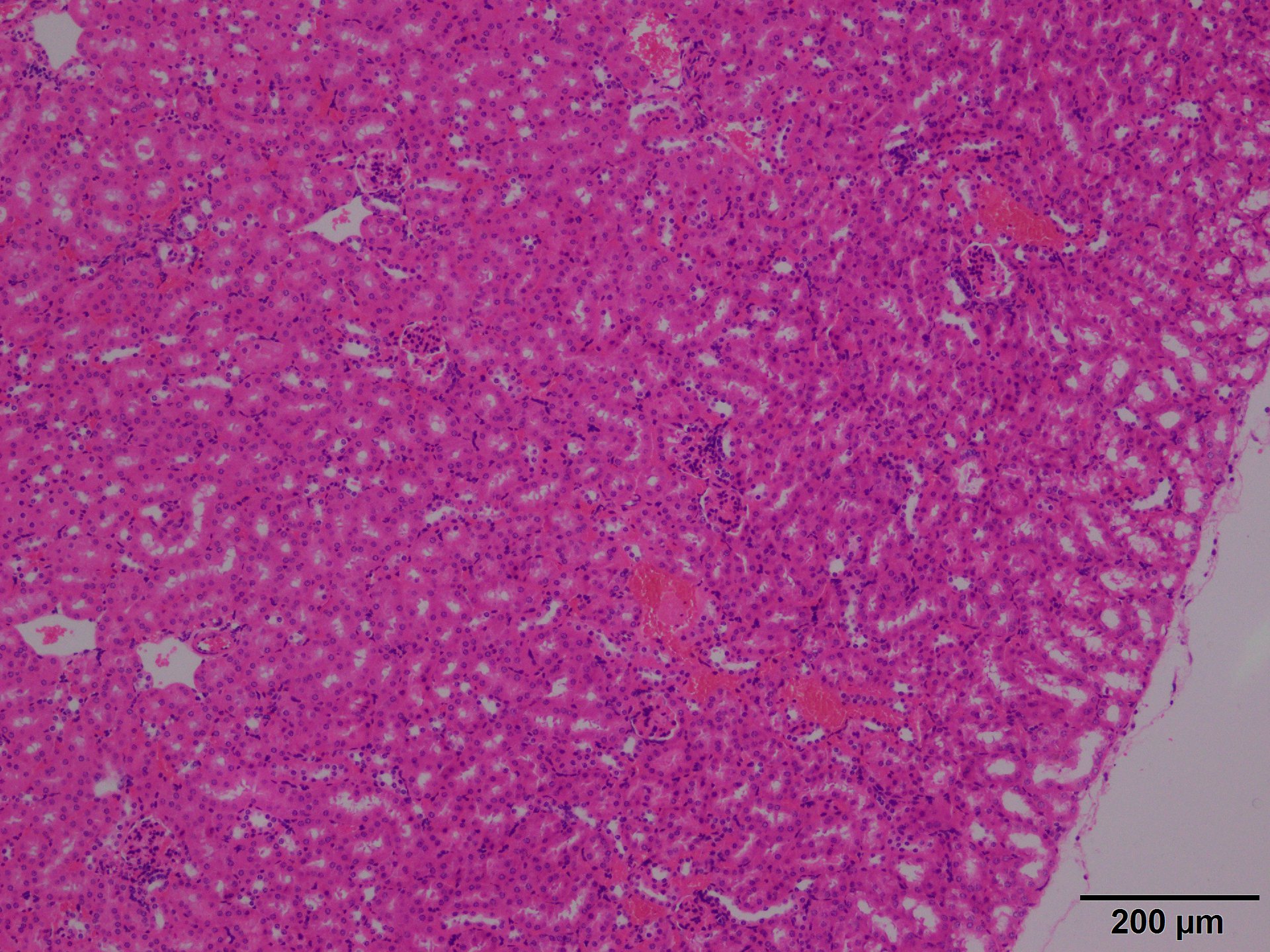

Supplement: Supplementary file 1 [file Data_Sheet_1.ZIP › Figure 4/LP-HFY05-H.jpg]

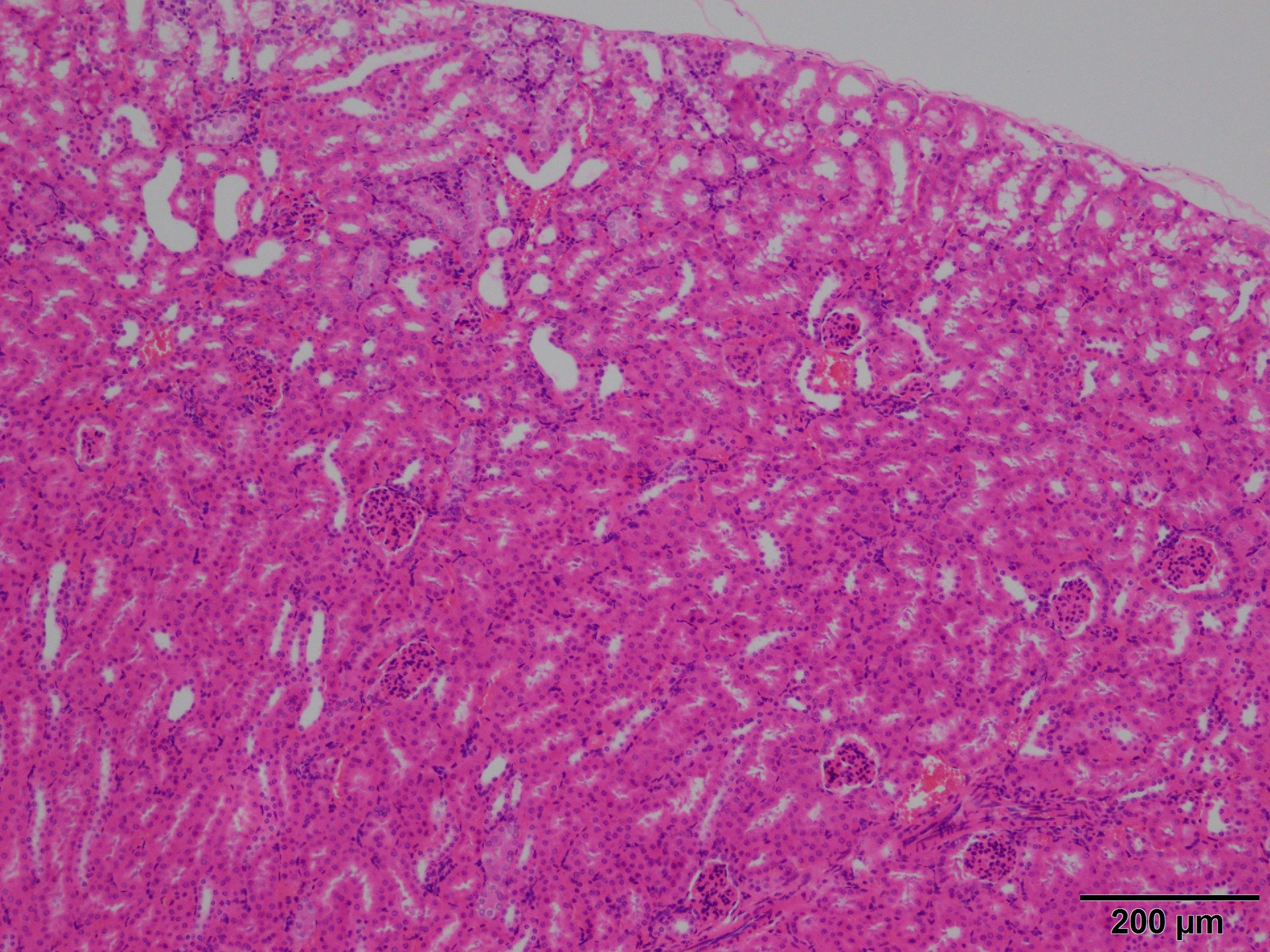

Supplement: Supplementary file 1 [file Data_Sheet_1.ZIP › Figure 4/LP-HFY05-L.jpg]

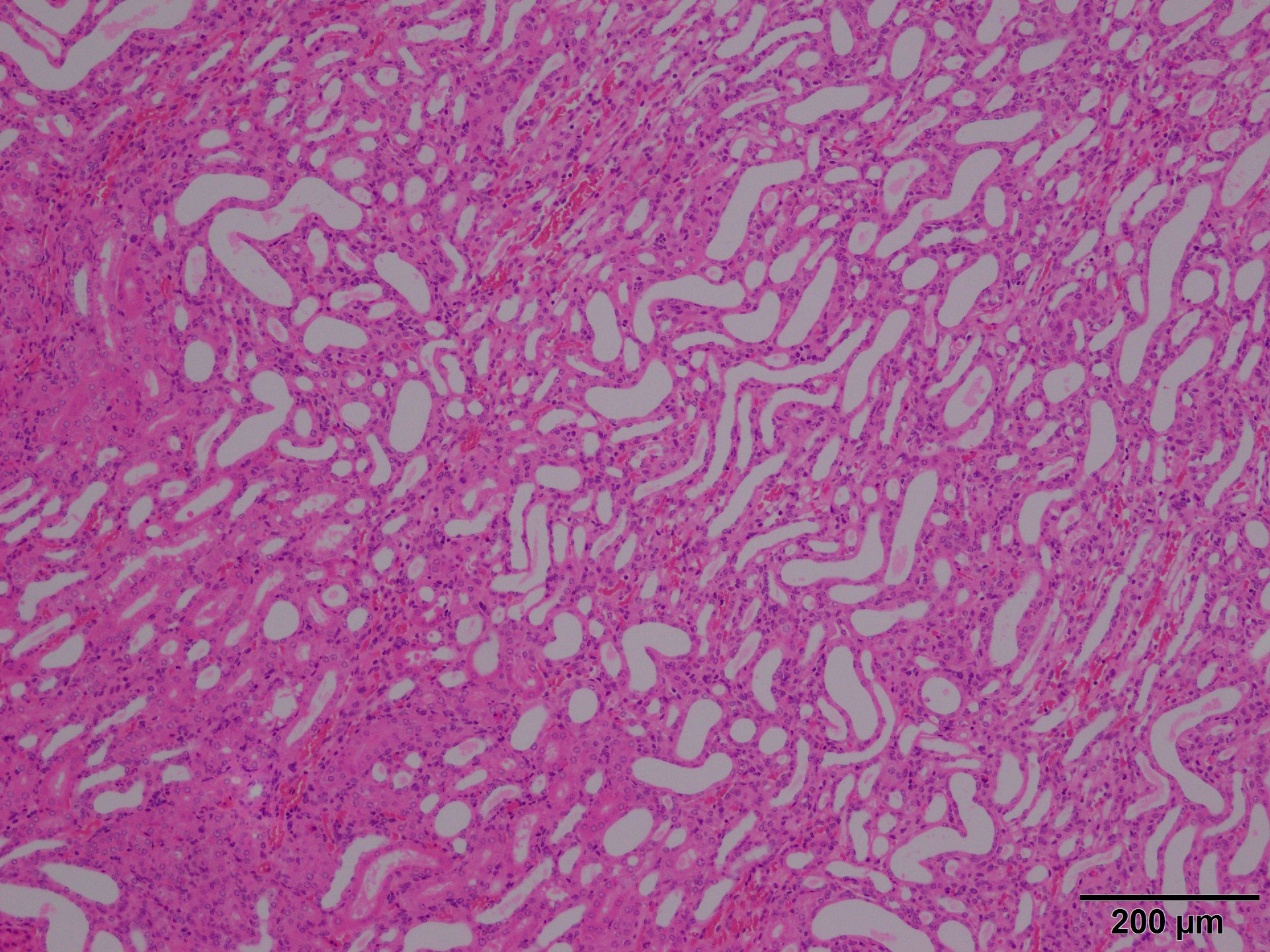

Supplement: Supplementary file 1 [file Data_Sheet_1.ZIP › Figure 4/model.jpg]

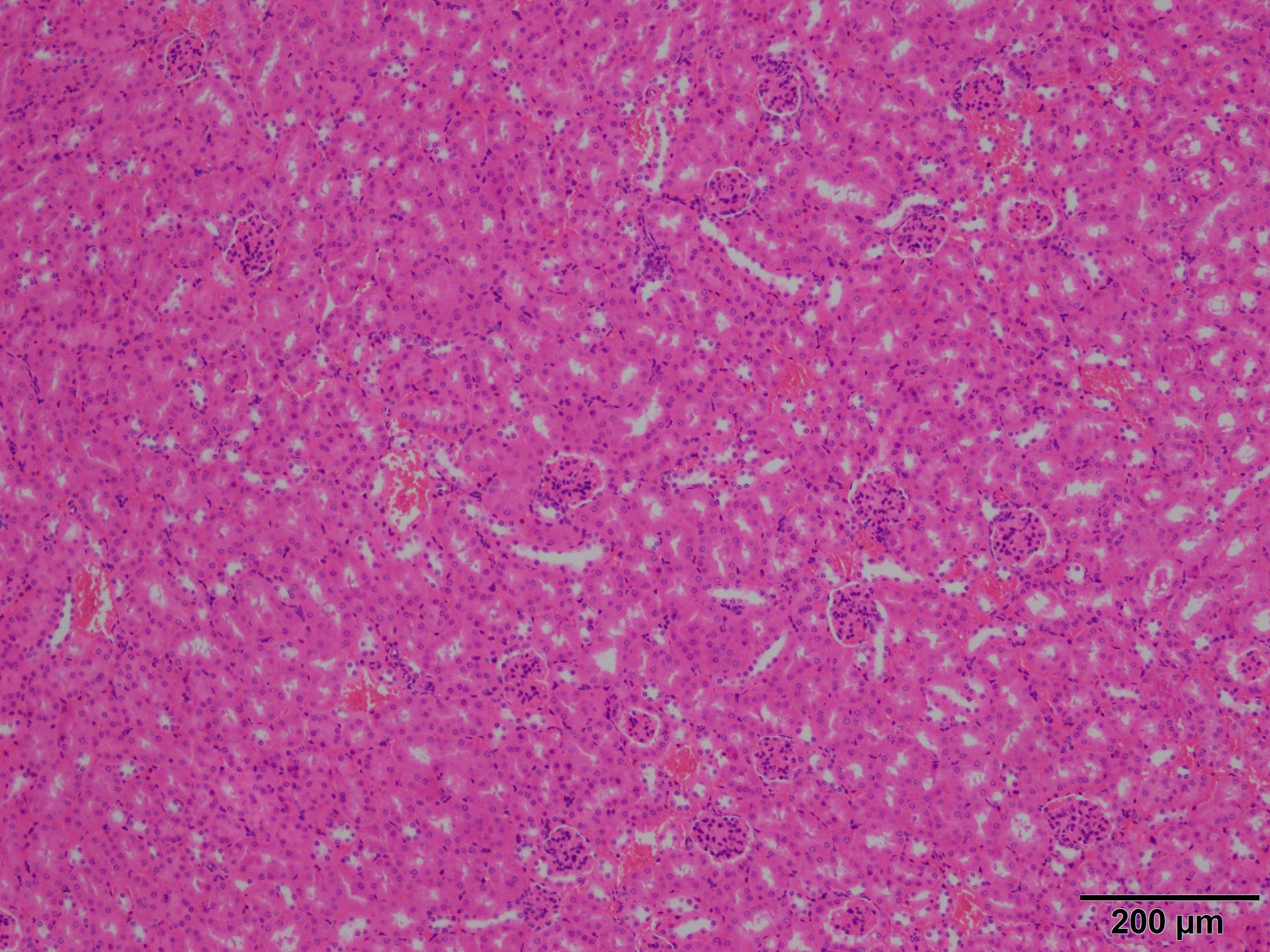

Supplement: Supplementary file 1 [file Data_Sheet_1.ZIP › Figure 4/normal.jpg]

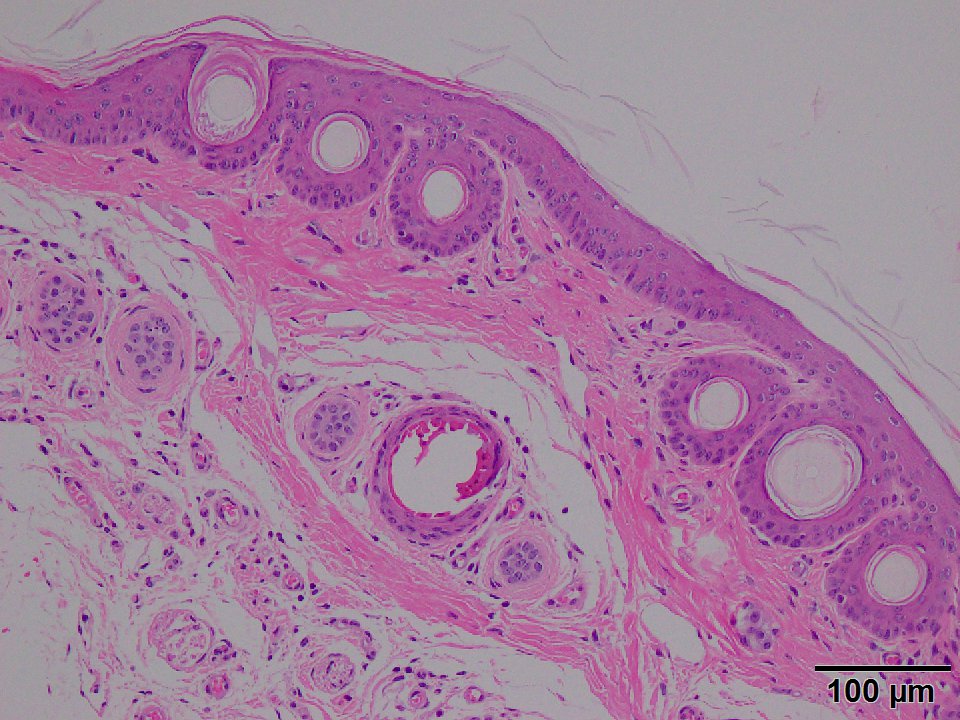

Supplement: Supplementary file 1 [file Data_Sheet_1.ZIP › Figure 5/dipyridamole.jpg]

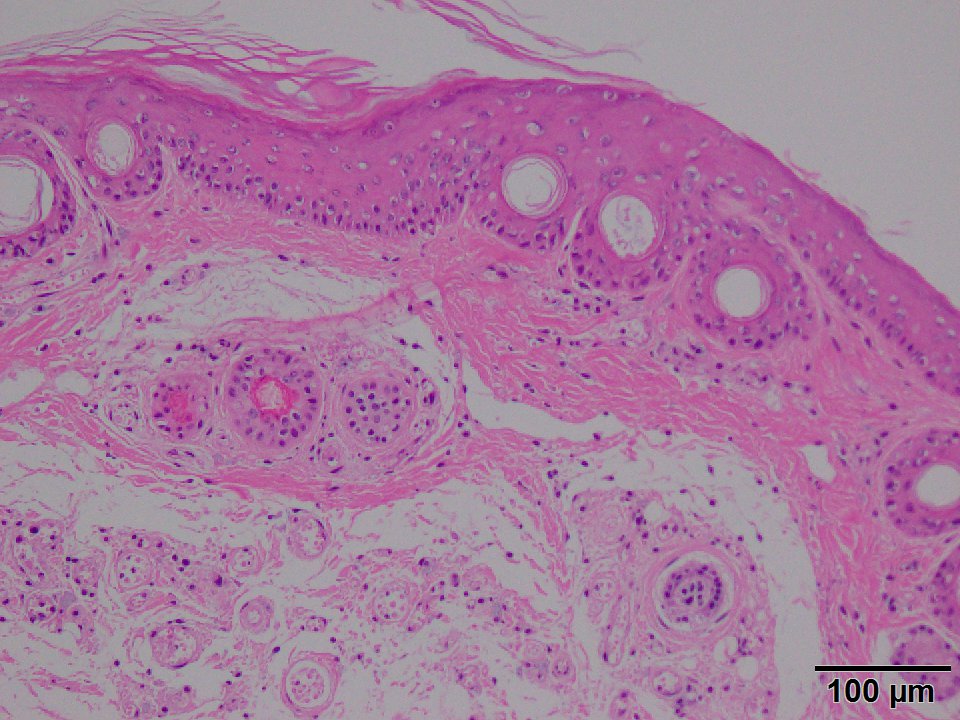

Supplement: Supplementary file 1 [file Data_Sheet_1.ZIP › Figure 5/LP-HFY05-H.jpg]

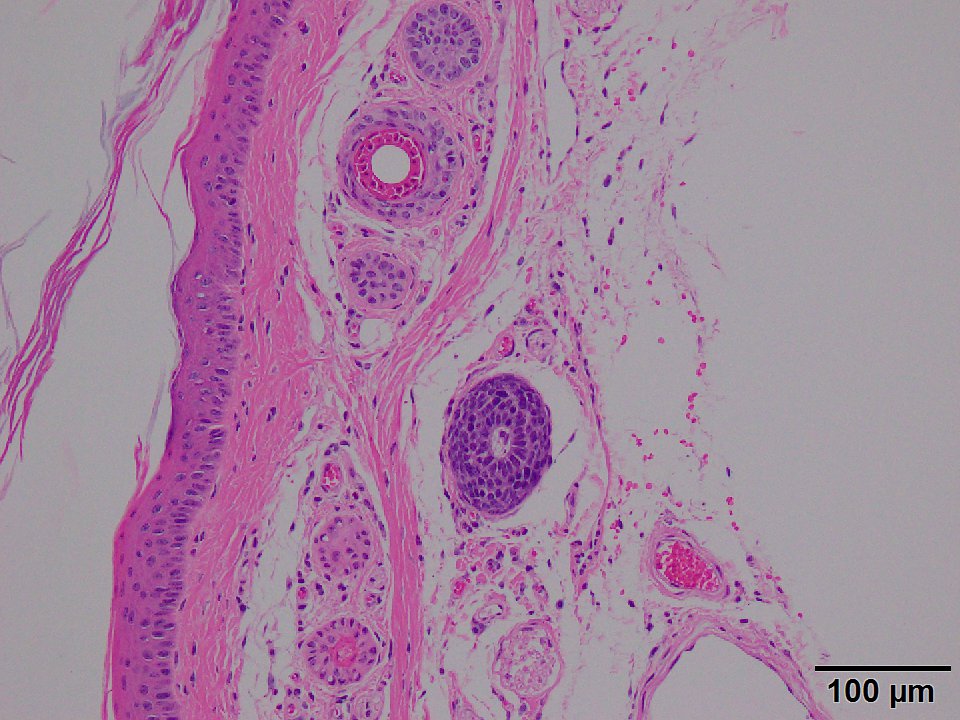

Supplement: Supplementary file 1 [file Data_Sheet_1.ZIP › Figure 5/LP-HFY05-L.jpg]

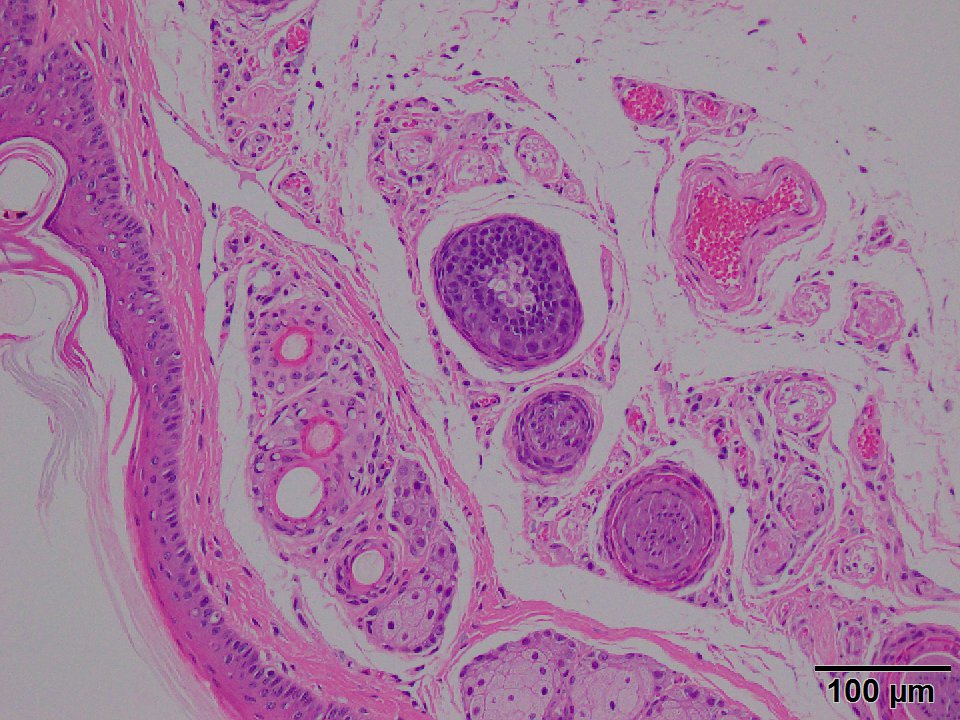

Supplement: Supplementary file 1 [file Data_Sheet_1.ZIP › Figure 5/model.jpg]

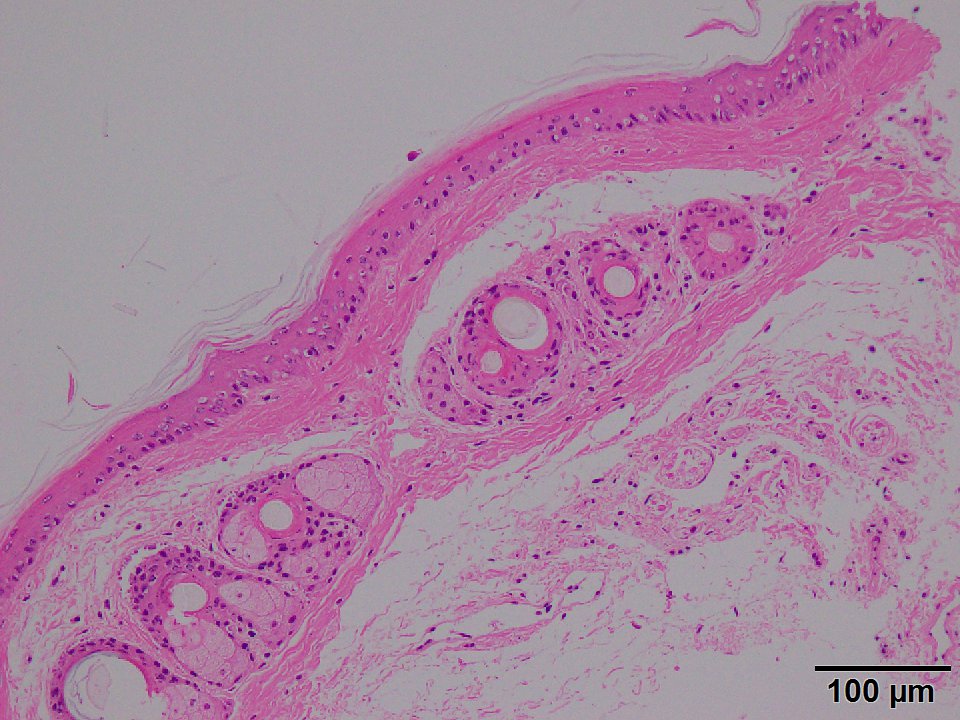

Supplement: Supplementary file 1 [file Data_Sheet_1.ZIP › Figure 5/normal.jpg]

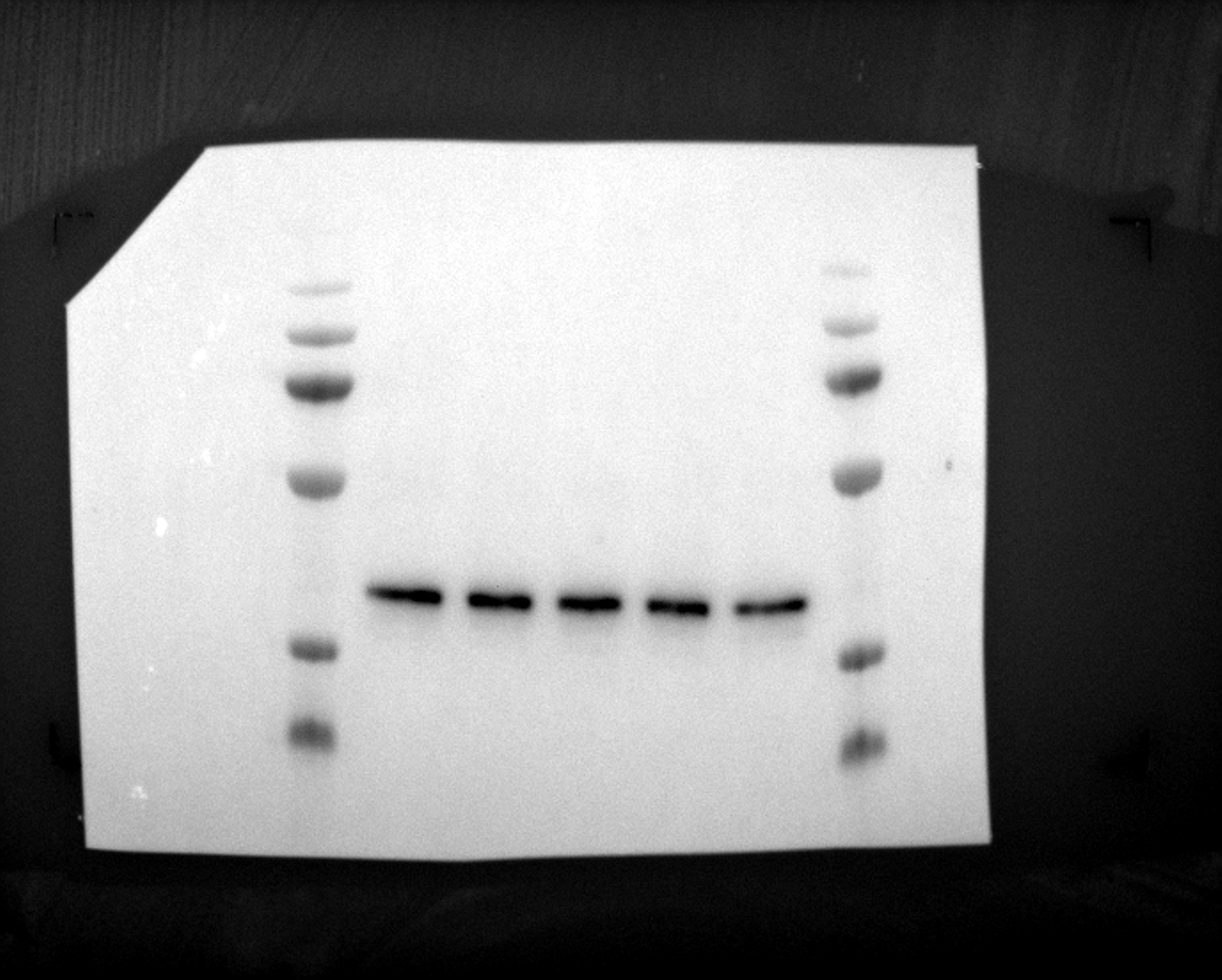

Supplement: Supplementary file 2 [file Data_Sheet_2.ZIP › β-actin-kidney.Tif]

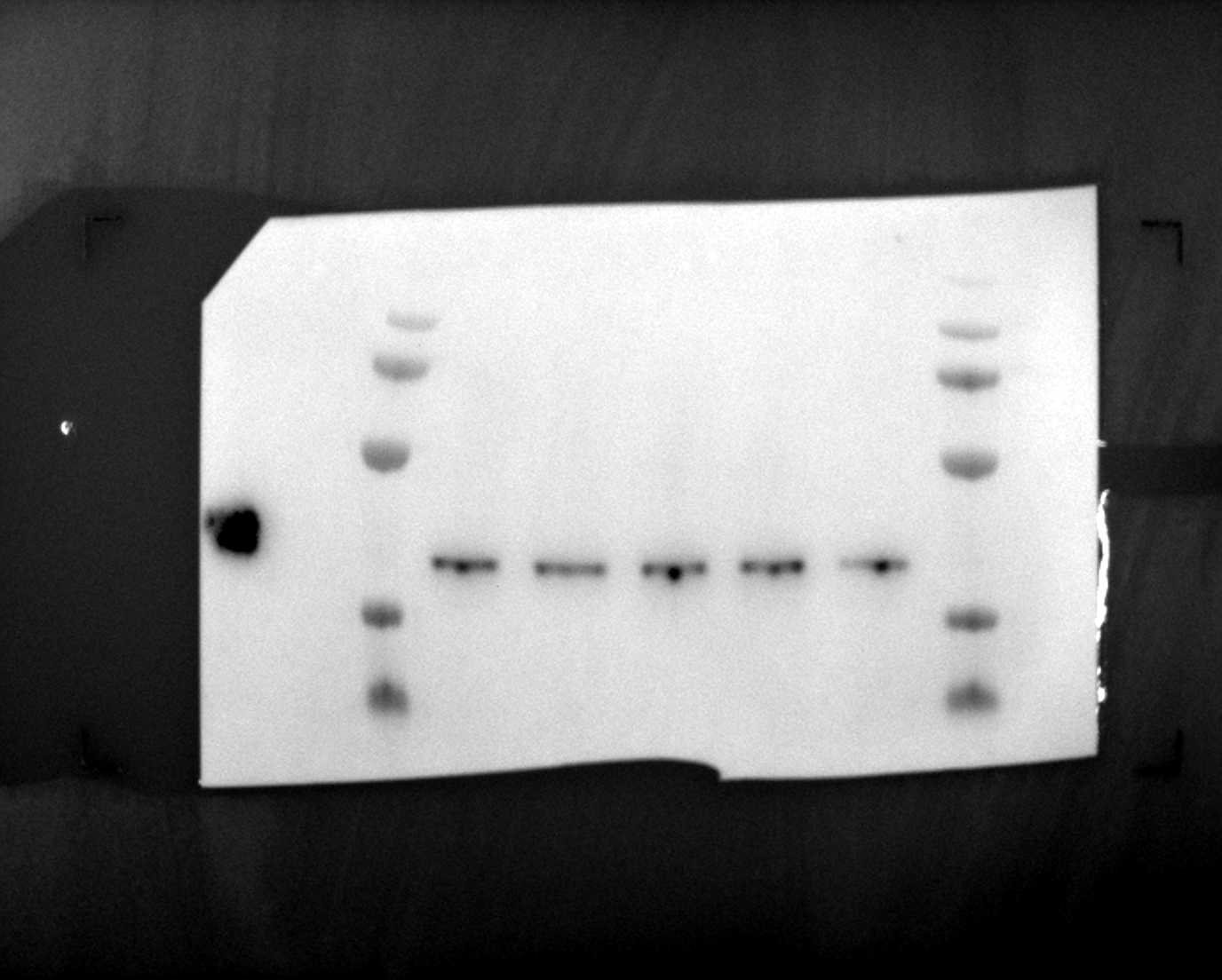

Supplement: Supplementary file 2 [file Data_Sheet_2.ZIP › β-actin-tail vein.Tif]

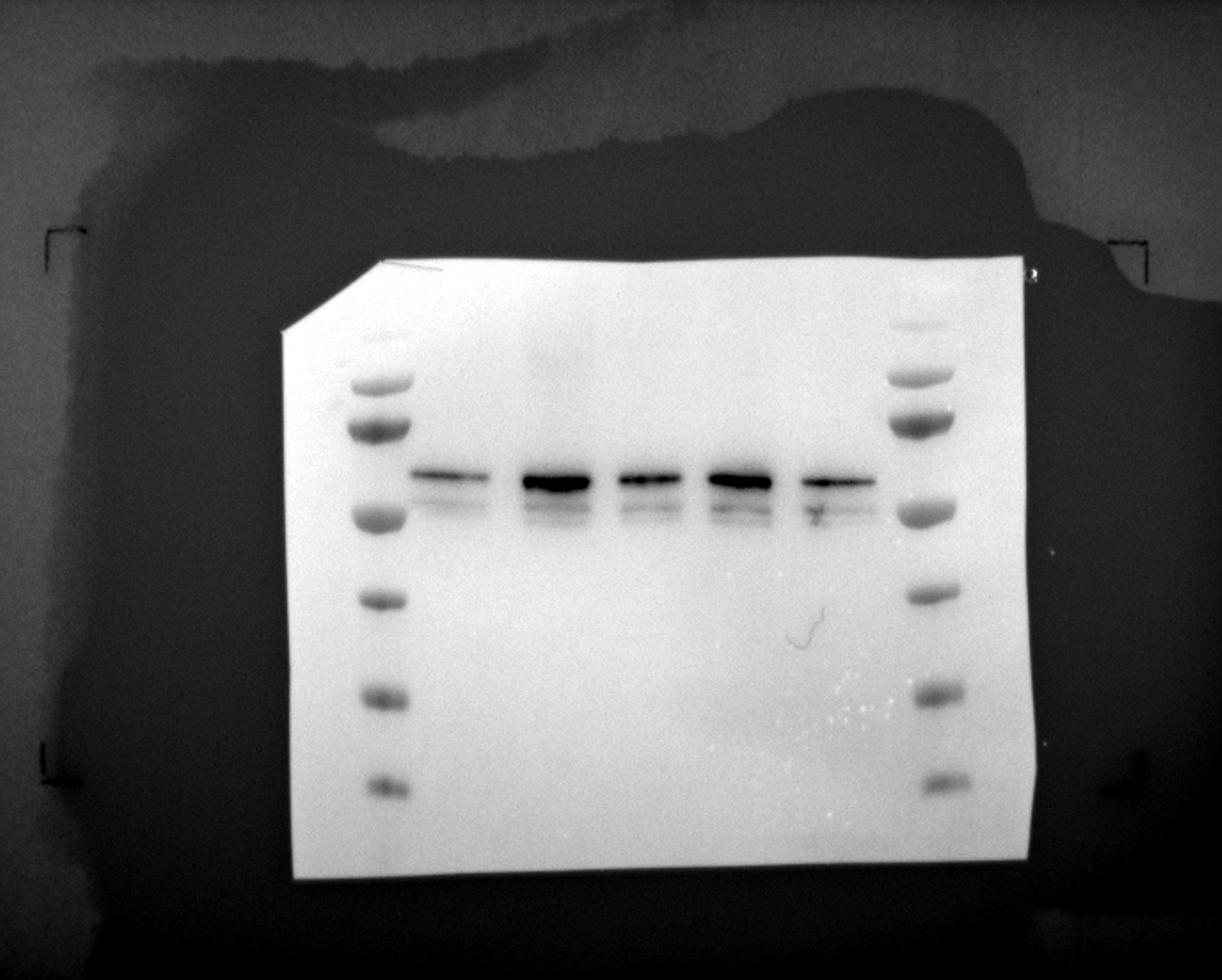

Supplement: Supplementary file 2 [file Data_Sheet_2.ZIP › NF-κB-kidney.Tif]

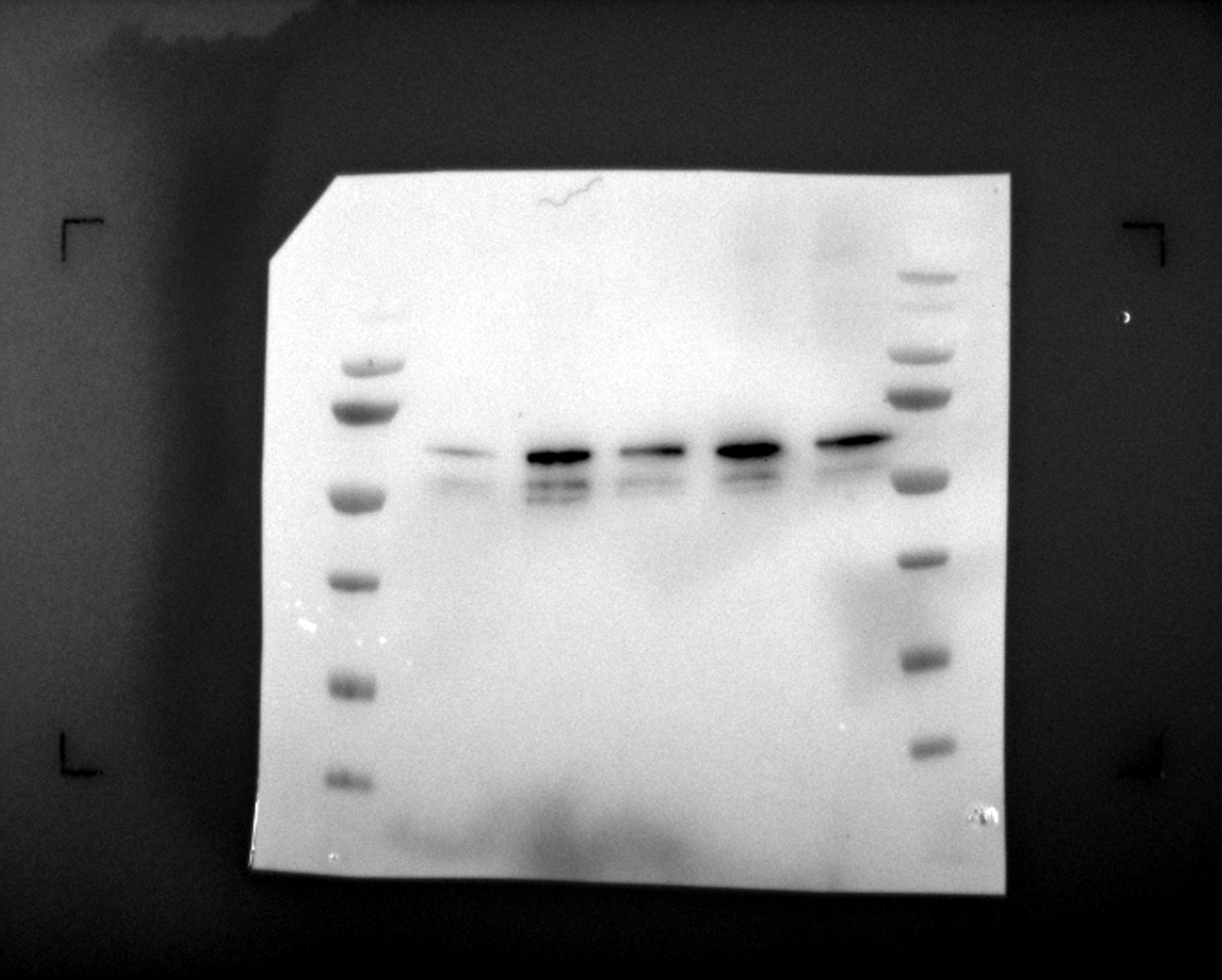

Supplement: Supplementary file 2 [file Data_Sheet_2.ZIP › NF-κB-tail vein.Tif]
